# Mechanical force-induced morphology changes in a human fungal pathogen

**DOI:** 10.1186/s12915-020-00833-0

**Published:** 2020-09-11

**Authors:** Charles Puerner, Nino Kukhaleishvili, Darren Thomson, Sebastien Schaub, Xavier Noblin, Agnese Seminara, Martine Bassilana, Robert A. Arkowitz

**Affiliations:** 1 Université Côte d’Azur, CNRS, INSERM, Institute of Biology Valrose (iBV), Parc Valrose, Nice, France; 2 Université Côte d’Azur, CNRS, Institute Physics of Nice (INPHYNI), Ave. J. Vallot, Nice, France; 3 Present Address: Manchester Fungal Infection Group, School of Biological Sciences, University of Manchester, Manchester, UK; 4 Present Address: Sorbonne University, CNRS, Developmental Biology Laboratory (LBDV), Villefranche-sur-mer, France

**Keywords:** Cell invasion, Mechanical force, Cell polarity, Cdc42, Cell morphology

## Abstract

**Background:**

The initial step of a number of human or plant fungal infections requires active penetration of host tissue. For example, active penetration of intestinal epithelia by *Candida albicans* is critical for dissemination from the gut into the bloodstream. However, little is known about how this fungal pathogen copes with resistive forces upon host cell invasion.

**Results:**

In the present study, we have used PDMS micro-fabrication to probe the ability of filamentous *C. albicans* cells to penetrate and grow invasively in substrates of different stiffness. We show that there is a threshold for penetration that corresponds to a stiffness of ~ 200 kPa and that invasive growth within a stiff substrate is characterized by dramatic filament buckling, along with a stiffness-dependent decrease in extension rate. We observed a striking alteration in cell morphology, i.e., reduced cell compartment length and increased diameter during invasive growth, that is not due to depolarization of active Cdc42, but rather occurs at a substantial distance from the site of growth as a result of mechanical compression.

**Conclusions:**

Our data reveal that in response to this compression, active Cdc42 levels are increased at the apex, whereas active Rho1 becomes depolarized, similar to that observed in membrane protrusions. Our results show that cell growth and morphology are altered during invasive growth, suggesting stiffness dictates the host cells that *C. albicans* can penetrate.

## Background

Polar tip growth, in which extension is limited to the apical surface, enables walled cells such as fungi and plants to explore their environment for nutrients and mating partners, while maintaining their surface to volume ratio [1]. Campas and Mahadevan [2] have derived simple scaling laws for cell geometry and identified a single dimensionless parameter that is sufficient to describe variation in the shape of tip growing cells using turgor pressure, cell wall elastic properties, and secretion rate. However, little is known with respect to the response of tip growing cells to mechanical stress. There are five fundamental types of mechanical stress: tension, compression, shear, torsion, and bending. Human and plant fungal pathogens can penetrate host tissue, and it is likely that they encounter compressive stress upon penetration and subsequent invasive growth. In addition to tissue penetration, growth within a spatially confined environment is critical for infection and successful dissemination of such fungal pathogens.

Fungal pathogens take advantage of different strategies to interact with their environment, of which tip growth is a common theme. Penetration of host tissue is critical for both human and plant fungal pathogens and requires not only the generation of sufficient force but also adhesion to the host cells to counter this force [3, 4]. Fungal pathogens have turgor pressures in the MPa range [3, 4], and for human fungal pathogens, this turgor pressure exceeds host cell resistance to penetration. Such host cells have elastic moduli that are in the 1–100 kPa range [5–7], although the critical stress for material rupture is determinant. Both the human fungal pathogens *Candida albicans* [8] and *Aspergillus fumigatus* [9] can actively penetrate host tissue, which is a critical step in the infection process [10–14]. Previous studies have revealed that *C. albicans* can invade cells via host-induced endocytosis and/or active penetration [8]. *C. albicans* invasion of the intestinal epithelia (small intestinal enterocytes) occurs almost exclusively by active penetration [8, 15, 16], whereas both endocytosis and active penetration are important for invasion of the oral epithelia [17]. However, even with oral epithelia, at the early stages of infection, active penetration is the major route for tissue invasion [17]. Hence, a better understanding of active penetration should provide insight into the initial step of tissue damage for mucosal infections. Translocation of *C. albicans* through intestinal epithelial layers is facilitated by the fungal peptide toxin candidalysin [16, 18]. Previous studies have shown that *C. albicans* hyphal tips are asymmetrically positioned during growth on a stiff surface, i.e., a “nose down” morphology, and that perpendicular growth and contact to a stiff topographical ridge (less than the hyphal radius) results in an indentation of the ridge [19].

To investigate the relationship between substrate stiffness and *C. albicans* penetration and invasive growth, we have used micro-fabrication, together with time-lapse microscopy. We show that there is a threshold for penetration that corresponds to a stiffness of ~ 200 kPa and that invasive growth within a stiff substrate is characterized by dramatic filament buckling along with a stiffness-dependent decrease in extension rate. Nonetheless, a small percentage of cells are able to invade 200 kPa PDMS, suggesting that these cells may play a key role in infection, similar to that of the persister cells in biofilms. Furthermore, we observed a striking alteration in cell morphology during invasive growth, which is not due to depolarization of active Cdc42, but rather occurs at a substantial distance from the site of growth, as a result of mechanical forces. Our data reveal that in response to mechanical forces, *C. albicans* has increased active Cdc42 at the apex while active Rho1 is depolarized, similar to what is observed in PDGF-induced fibroblast membrane protrusions [20].

## Results

### Monitoring *Candida albicans* filamentous growth in micro-fabricated chambers

**Fig. 1:**
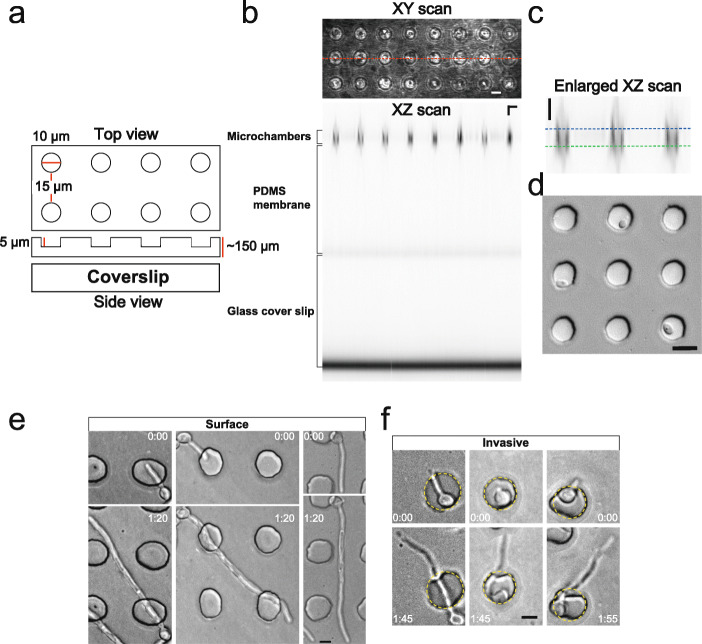
Filamentous growth in and on PDMS. **a** Schematic of PDMS microchamber array. Top and side views are shown with dimensions indicated. **b** Characterization of PDMS microchamber array. XY transmission view (top) with the location of XZ confocal reflection scan (bottom) of upright PDMS mounted on a coverslip (indicated by dotted red line). Bars are 10 μm in XY and XZ. **c** Enlarged XZ scan of PDMS microchambers. XZ confocal reflection microscopy of 3 microchambers with dotted lines, indicating top and bottom. Bar is 10 μm. **d** PDMS microchambers with *C. albicans* cells entrapped. *C. albicans* cells were mixed with serum (75%) and added to PDMS microchamber arrays, and DIC image is shown. Note the cell with a germ tube (top row). Bar is 10 μm. **e** Non-invasive filamentous growth on PDMS micro-arrays. DIC images of growth on PDMS surface from 3 independent experiments on 40:1 PDMS to cross-linker, with indicated times (h:min). **f** Invasive filamentous growth within PDMS microarrays. DIC images of growth within PDMS from 2 independent experiments using 40:1 PDMS to cross-linker with times indicated. The initial PDMS chamber is outlined with a dotted yellow line to highlight deformation upon invasive filament extension

**Fig. 2:**
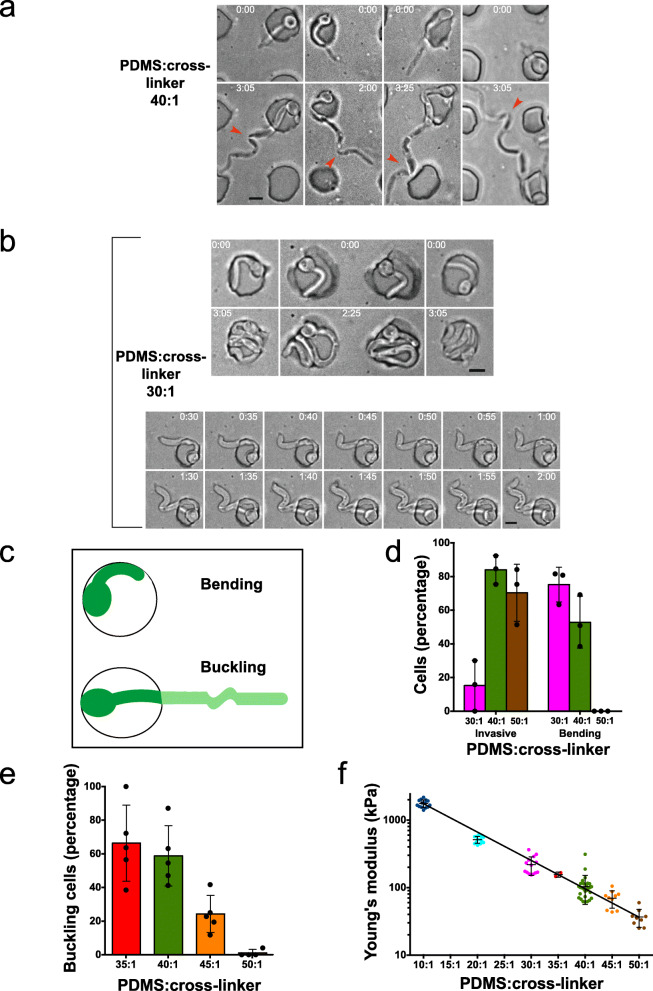
Filament subapical bending or buckling during invasive growth or confinement in PDMS chamber. **a** Filament buckling during invasive growth. DIC images of representative cells growing within PDMS at indicated times; red arrowheads indicate filament buckling. **b** Filament buckling upon confinement in stiff PDMS chambers. Cells entrapped in the PDMS chamber at indicated times visualized by DIC (top panel). Time-lapse of a cell grown on PDMS, probing the surface (bottom) followed by filament buckling and release, at indicated times. **c** Schematic of bending and buckling cells. Top cell bending in the microchamber and bottom filament buckling within PDMS (lighter green indicates part within PDMS). **d** PDMS invasion and filament bending are inversely correlated. Three independent time-lapse experiments were carried out at indicated PDMS to cross-linker ratios. Filament curvature upon contact or penetration of PDMS was scored as bending (bending or buckling within PDMS was not scored). Bars indicate SD, and points indicate experimental mean (*n* = 20–60 cells per experiment and 90–140 per condition). **e** Filament buckling increases with increasing PDMS stiffness. Independent time-lapse experiments (4–5) were carried out at indicated PDMS ratio, with *n* = 20–50 invasive cells per experiment (90–125 per condition). Bars indicate SD, and points indicate experimental means. Invasive filaments were considered buckling if the angle of the filament was 90° or greater, compared to filament tip. Note that no buckling was observed in filaments growing on the PDMS surface. **f** Young’s modulus of PDMS preparations. Young’s modulus was determined by a viscoanalyzer for 10–30 preparations (except 35:1, only 2 preparations) at indicated PDMS to cross-linker ratios

To investigate *C. albicans* hyphal growth, we took advantage of micro-fabrication approaches using the elastomer polydimethylsiloxane (PDMS) that, in particular, have been reported as single-cell force sensors for fission yeast cells [21]. We generated PDMS arrays with approximately 10^5^ microchambers, which were cylindrical in shape with a diameter of 10 μm, a depth of 5 μm, and 15 μm spacing between adjacent chambers (Fig. 1a). *C. albicans* cells in micro-fabricated PDMS chamber arrays were visualized with inverted microscopes; imaging was carried out through an upright array of 150–200-μm-thick PDMS. Figure 1b shows an XZ confocal reflectance scan through the PDMS microarray with the chambers and media at the top (highest position) and the coverslip below for support (a zoom of chambers is shown in Fig. 1c). *C. albicans* cells were mixed with fetal calf serum, added to the PDMS array, incubated for ~ 1 h, and subsequently, filamentous growth was followed over time (Fig. 1d). With low-stiffness PDMS (a high polymer to cross-linker ratio of 40:1) we observed two predominant filamentous growth modes: non-invasive growth on the PDMS surface and invasive growth within PDMS (Fig. 1e, f). By examination of the focal plane of the PDMS surface and the fungal filaments, using DIC optics, we were able to distinguish between non-invasive (surface) and invasive growth, referring to whether the filament tip is on or within the PDMS, respectively. Invasive growth was also confirmed by labeling the PDMS surface and filamentous cells (see below). Furthermore, we observed that the blastospore (round cell) portion of the filamentous cells, which grew in the microchambers, pushed back against the chamber wall upon PDMS filament penetration and the filament frequently buckled within PDMS, presumably due to the resistive force during growth within the elastomer (Figs. 1f and 2a). These results indicate that, in addition to having ideal optical properties, PDMS is compatible with *C. albicans* filamentous growth.

### Growth modes depend on substrate stiffness

We followed *C. albicans* filamentous growth in PDMS of different stiffness, i.e. the extent to which an object resists deformation in response to an applied force, by varying the ratio of polymer to cross-linker. We observed two main growth modes from cells initially in chambers depending on PDMS stiffness: invasive growth, which predominated with less stiff PDMS (40:1) (Fig. 2a) and dramatic bending in the stiffer PDMS (30:1) chambers, which was predominantly subapical (Fig. 2b, top panel). In contrast to *Schizosaccharomyces pombe* [21], extensive deformation of the chambers was not observed with *C. albicans*, which we attribute to the different sizes, geometries, and growth modes of these fungi; fission yeast has a radius of ~ 2 μm, compared to *C. albicans* hyphal filaments with a radius of ~ 1 μm [22] (and our observations), resulting in a greater than 4-fold difference in the cross-sectional area. At most, a slight chamber deformation was observed with *C. albicans*, as cells frequently popped out of the chambers comprised of stiff PDMS or penetrated this material, when less stiff. Occasionally, at intermediate PDMS stiffness, we observed filamentous cells growing on the PDMS surface that appeared to be probing the surface with a “nose down” growth (Fig. 2b, bottom panel), as similarly observed [19]. This type of growth was suggestive of the filaments attempting to penetrate into PDMS and, consistent with this, we observed these filaments buckling and/or bending subapically at each attempt, prior to the tip popping out and forward. Buckling is defined as a sudden change in the shape of a component under load, i.e. change in the shape of the filament due to the physical forces it experiences. Subapical bending is, additionally, defined as a change in the direction of growth that results in curved filaments (Fig. 2c). In buckling, it is expected that the shape changes are largely reversed upon removal of the external forces, whereas in bending, the shape changes are not a result of the mechanical forces directly. Buckling can occur with a filament initially straight or bent/curved.

We next examined whether cells, initially in chambers, which were unable to invade, underwent bending. Figure 2d shows that as the stiffness of PDMS increased (from 50:1 to 30:1 PDMS to cross-linker), there was an increase in the percentage of cells undergoing bending, concomitant with a decrease in those invading PDMS. During invasive growth, we also frequently observed buckling of the filament (Fig. 2a red arrowheads, c), i.e. a growth-dependent curvature that typically occurred at the portion of the filament within PDMS. Figure 2e shows that such buckling was dependent on the PDMS stiffness, with over half of invasive filaments buckling in the two stiffest PDMS (40:1 and 35:1).

To determine the mechanical properties of the different PDMS preparations, we used dynamic mechanical analysis for which measurements were reproducible over a range of PDMS stiffness. Oscillating strain at a frequency of 10 Hz was applied to PDMS samples, and stress (*σ*)-strain (*ε*) curves were obtained (Additional file 1: Figure S1), from which Young’s modulus was determined (initial *dσ*/*dε*). Young’s modulus is the quantitation of the stiffness, i.e. the ratio stress/strain for a uniaxial load, where stress is the force per unit area and strain is the proportional deformation (change in length divided by original length) and is dimensionless. Figure 2f shows the Young’s modulus of different ratios of PDMS to cross-linker, which are in good agreement with published values [23–26]. The stiffness of lower ratio samples (10:1 and 20:1), intermediate ratios (30:1 to 40:1), and higher ratios (45:1 and 50:1) was similar to that of medical silicone implants [27], with a Young’s modulus of ~ 1 MPa; stiff tissues such as the myocardium [7], with a Young’s modulus of ~ 0.1–0.2 MPa; and less stiff tissues such as the epithelia [5, 6], with a Young’s modulus of ~ 40–70 kPa, respectively.

### Penetration into and escape from PDMS

**Fig. 3:**
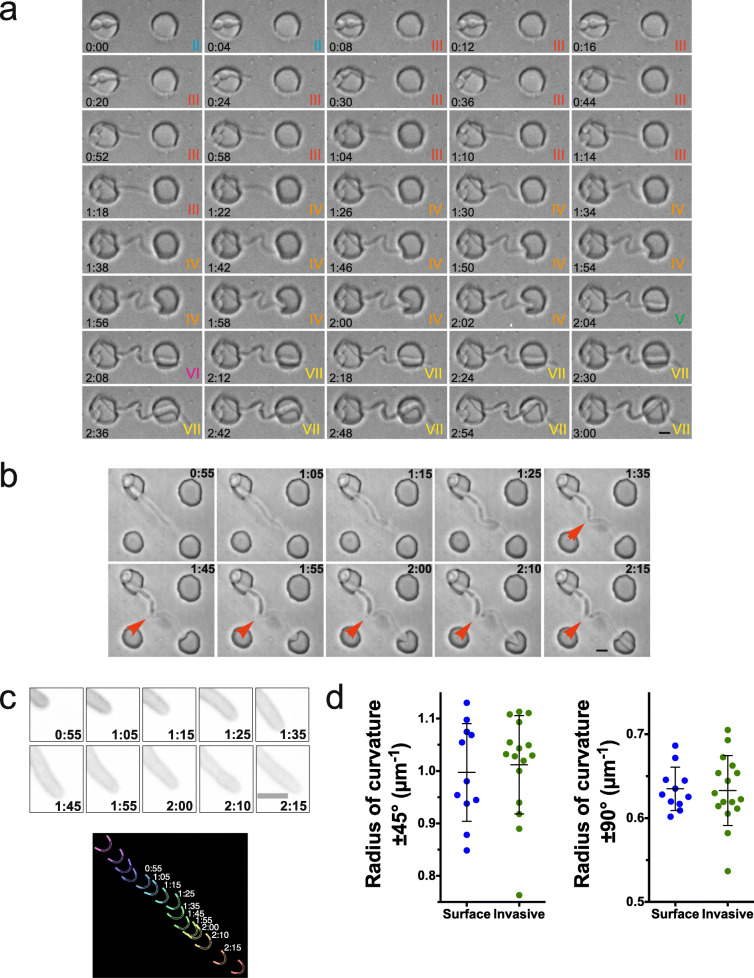
The filament tip shape is not substantially altered upon invasive growth and burst out. **a** Filament buckling and release upon invasive growth and penetration. Time-lapse experiment at PDMS to cross-linker ratio of 40:1 (Young’s modulus ~ 100 kPa) with DIC images acquired every 2 min. **b**, **c** Filament tip shape does not substantially change during PDMS burst out. **b** Typical time-lapse experiment at 40:1 PDMS to cross-linker ratio (Young’s modulus ~ 100 kPa) with DIC images every 5 min. Red arrowheads indicate filament buckling. **c** Close up of sum projections of 13 × 0.5 μm *z*-sections GFP-Ct_Rac1_ fluorescent images from 3B (top), with tip curvature (bottom) at indicated times and ± 45° curvature indicated by open lines and ± 90° indicated by solid lines. **d** The radius of the curvature of invasive and surface growing cells is indistinguishable. The radius of the curvature is the average of 12–24 × 5 min time points (*n* = 11–16 cells) from 3 to 4 independent experiments for surface (PDMS ratio 40:1; Young’s modulus ~ 100 kPa) or invasively growing cells (with PDMS ratio 35:1–40:1; Young’s modulus 150–100 kPa). Bars indicate SD

Given that active penetration is critical during the process of *C. albicans* epithelium invasion [10–12, 14], we examined in further detail this process in PDMS. Figure 3a shows a filamentous cell that penetrates PDMS after 4 min (II; note that I, not shown, is prior to the filament contacting the chamber wall); subsequently grows invasively within PDMS (III); deforms the adjacent chamber (IV), resulting in a dramatic invagination; and exits PDMS into the adjacent well at 2:04 (V), followed by penetration into the opposing chamber at 2:08 (VI) and subsequent invasive growth (2:12; VII). The resistive force revealed by buckling of the filament, as well as deformation of the initial chamber during invasive growth (III), likely increases upon deformation and subsequent piercing into the adjacent well (IV), as the portion of the filament within PDMS buckled during this time (1:22–2:02), resulting in an S-shaped filament (Fig. 3a). The tension on the filament was released upon exiting PDMS into the adjacent well (V), as the tip of the filament appears to jump forward (2:04). The resistive force from the final step of growth (VII) also resulted in buckling of the filament (portion in the well) leading to an M shape (2:42–3:00). This escape from PDMS is analogous, in some respects, to filaments bursting out of a macrophage [28–31]. Here, the filament pushes into a circle resulting in a deformation that does not require expansion of the surface area but rather local invagination of the chamber, which is easier to detect (Fig. 3a). Indeed, such a bursting out of PDMS was observed a number of times, and Additional file 1: Figure S2 shows such examples in different PDMS stiffness (40:1, 110 kPa; 35:1, 150 kPa; and 30:1, 250 kPa).

**Fig. 4:**
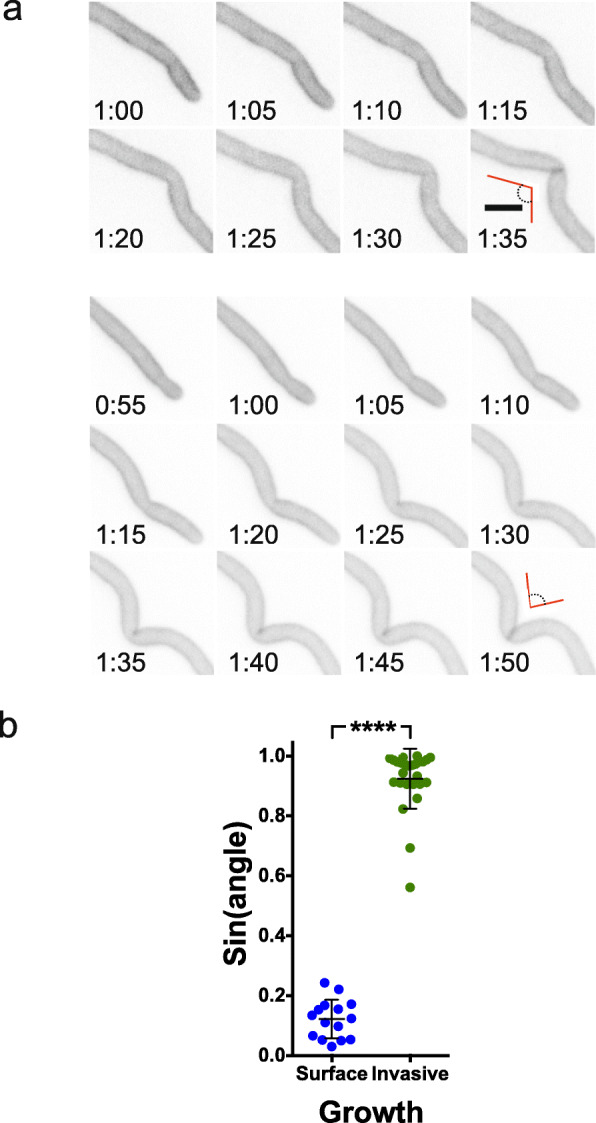
Cell division occurs at the site of filament buckling during invasive growth. **a** Examples (upper and lower panels) of time-lapse images (GFP sum projections) during invasive growth in 40:1 PDMS ratio, showing cell division site at the location of filament buckling with red lines highlighting the buckling angle. **b** Cell division site occurs where the filament is buckled. Cells were grown either invasively within PDMS (40:1; Young’s modulus ~ 100 kPa) or on the surface of PDMS (30:1–40:1; Young’s modulus 250–100 kPa), *n* = 26 and 15, respectively. GFP sum projections were analyzed over time from 5 independent experiments and Sin of angle when the septum formed is shown. Septum formation was observed on average 47 ± 20 min after buckling was evident in invasive cells. Bars indicate SD with *p* < 0.0001

In order to better visualize the invasive growth within PDMS during these different steps, we followed cells in which GFP was targeted to the plasma membrane [32], by confocal spinning disk microscopy acquisition over a range of *z*-positions. Figure 3b and c show a typical time-lapse acquisition in which the analysis of the cell outline did not reveal a substantial change in the shape of the filament tip during invasive growth and bursting into the next well (Fig. 3c, d; Additional file 1: Figures S3A and S3B). Indeed, the radius of the curvature of the cell tip was identical to that of surface-growing cells, and there were no changes upon burst out of PDMS. Buckling of the filament was evident upon invasive growth and occurred over 35–45 min, prior to the appearance of a septum (Fig. 3b (red arrowheads) and Fig. 4a, two examples). Analyses of the angle of the filament at which the septum formed ultimately, indicate that during invasive growth, cytokinesis occurs the majority of the time after the filament buckles (Fig. 4b).

**Fig. 5:**
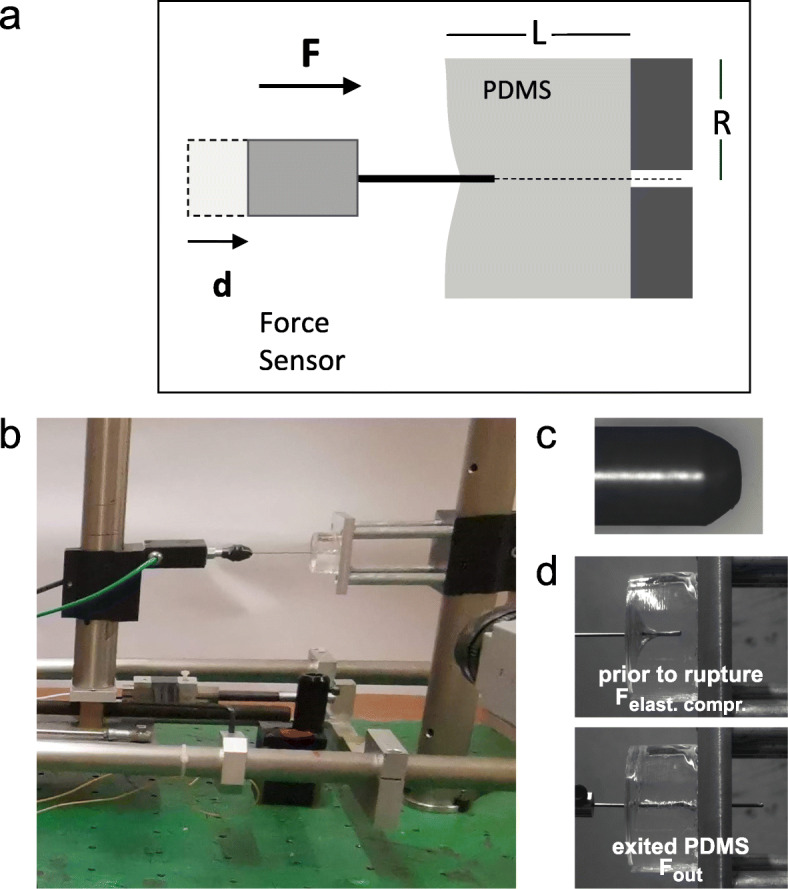
Physical model of PDMS invasive growth. **a** Schematic of the physical model. The distance probe traveled is *d*; *F* is the force detected by the sensor; *L* is the PDMS length, and *R* is the PDMS radius. **b** Image of the physical model setup. Force sensor attached to 1-mm-diameter probe (left) and PDMS on the right as indicated in **a**. **c** Shape of the steel probe tip. Probe is 1 mm diameter. **d** PDMS penetration. PDMS (40:1; Young’s modulus ~ 100 kPa) cylinder (1.75 cm length and radius) with 1 mm probe displacement of 1.6 μm/s. Upper image, prior to rupture of PDMS; lower image, probe exit from PDMS

**Fig. 6:**
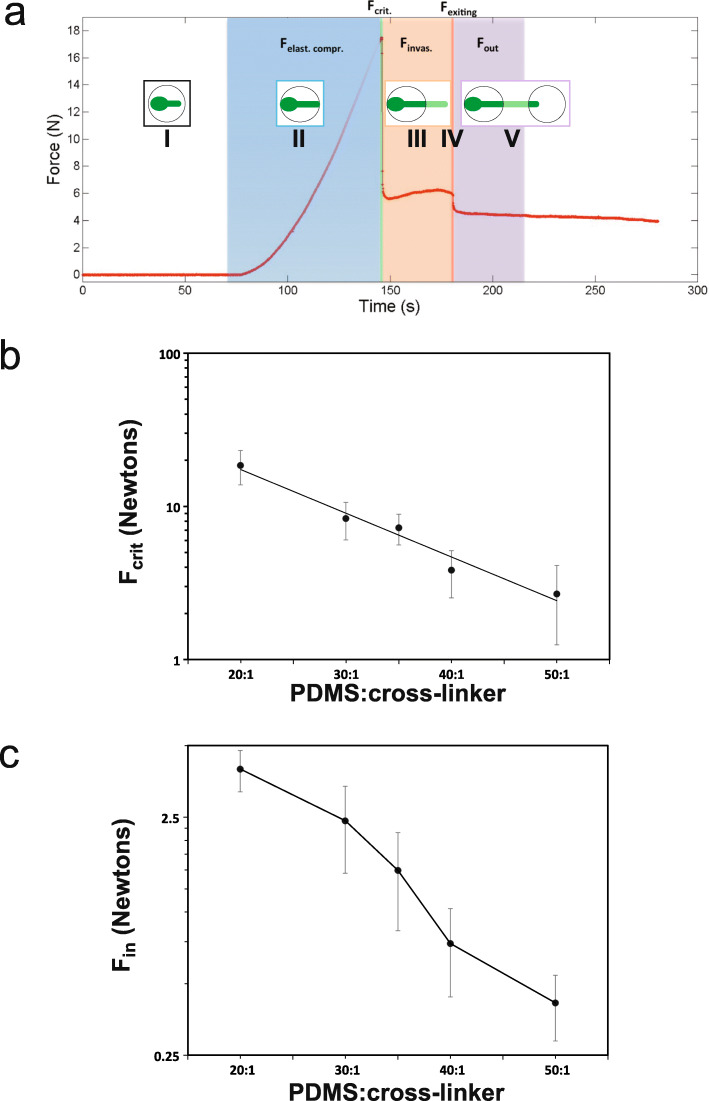
Forces in physical model of PDMS invasive growth. **a** Forces encountered upon probe displacement into and out of PDMS. A 1-mm-diameter steel probe was advanced perpendicularly into the base of the cylindrical piece of cured PDMS (10:1 PDMS to cross-linker ratio, Young’s modulus ~ 2 MPa). Probe displacement rate was 63 μm/s, and force was determined during different stages, as indicated, i.e., compression, penetration, and after the probe tip emerged from PDMS. Analogous stage of hyphal filament invasive growth indicated in insets along with the growth stage from time-lapse in Fig. 3a (I–V). **b**, **c** Resistive forces as a function of PDMS to cross-linker ratio. **b** *F*_crit_ was determined using a 1-mm-diameter steel probe, with either 3.5- or 8-cm-diameter PDMS cylinders of indicated PDMS to cross-linker ratio, with probe displacement set to 1.6–3.2 μm/s. Bars are SD for on average 10 independent determinations for each PDMS ratio. **c** *F*_in_ was determined by subtracting *F*_out_ from *F*_invas_ from the probe penetration experiment described in **a**. Bars are SD

To analyze the physical constraints during penetration, invasive growth, and tip escape from the PDMS matrix, we established a physical experimental model, which consisted of a steel probe that mimics the filament shape, continuously advancing up to and into a cylinder of PDMS of different stiffness (Fig. 5a, b). The steel probe tip approximated the shape of the filament tip (Fig. 5c) with a radius of curvature (± 45°) of 1.1 μm when normalized to the hyphal filament, compared to that of 1.0 ± 0.1 μm for the surface and invasively growing hyphal filaments. Figure 5d shows the probe prior to PDMS rupture and subsequent to exiting from the PDMS. Experiments were carried out with a range of probe displacement rates encompassing that of the filament extension rates (~ 0.3 μm/min [33]), when scaled down to the filament diameter. Figure 6a shows an example of such a force versus displacement curve. The initial phase of increasing force corresponds to the elastic compression of PDMS (*F*_elast compr_; analogous to growth stage II, Fig. 3a), culminating in the force required to break the PDMS surface, *F*_crit_. The next phase corresponds to the extension within PDMS, analogous to invasive growth, *F*_invas_ (analogous to growth stage III, Fig. 3a) culminating in PDMS exit (*F*_exiting_; analogous to the end of growth stage IV, Fig. 3a). The final phase corresponds to when the end of the probe has emerged from the PDMS (*F*_out_; analogous to growth stage V, Fig. 3a). Figure 6b shows the *F*_crit_ from the physical probe experiments as a function of PDMS stiffness. Filaments are able to penetrate PDMS of ratio 35:1, for which we measured *F*_crit_ of ~ 7 N with a 1-mm-diameter probe. Scaled to the diameter of a hyphal filament, this would correspond to a force of ~ 31 μN, indicating that hyphal filaments generate forces larger than 31 μN to penetrate PDMS. Scaling to the diameter of a hyphal filament was done assuming a constant critical stress, given the filament diameter is several orders of magnitude greater than the PDMS mesh size, and was calculated by taking the ratio of the metal probe/filament radius squared. Furthermore, our results indicate that at a stiffness of 200 kPa, for which little PDMS penetration is observed (Fig. 2d), the *F*_crit_ is ~ 8 N with a 1-mm-diameter probe, i.e. ~ 35 μN when scaled to a hyphal filament, which would correspond to the growth stalling force.

### Resistive force affects hyphal extension and morphology

**Fig. 7:**
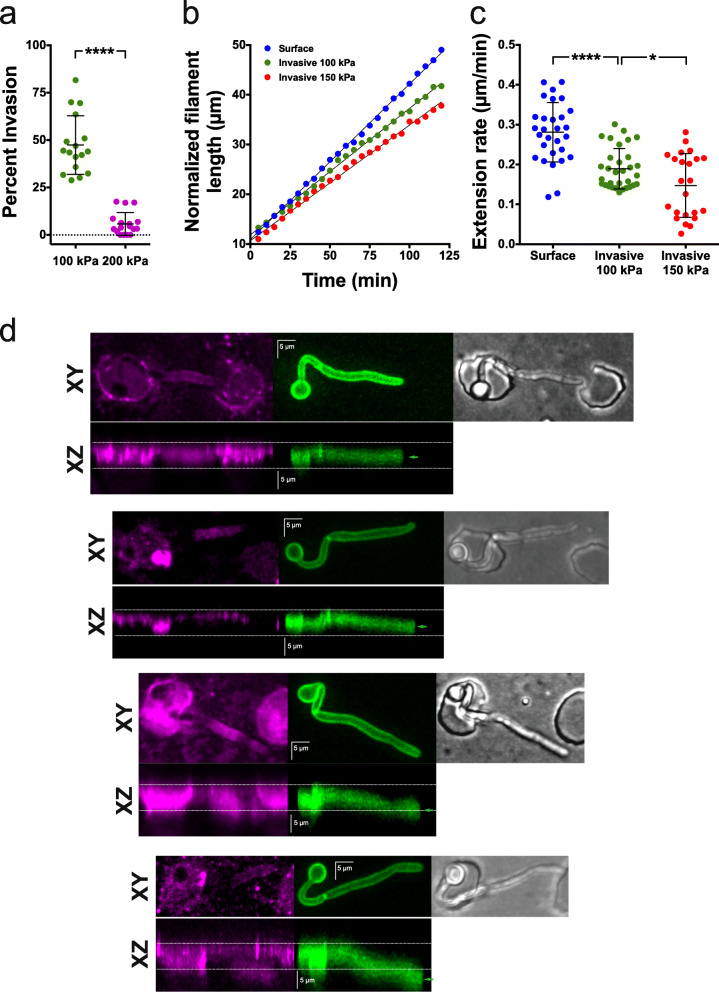
Filament extension rate is decreased during invasive growth. **a** A PDMS substrate stiffness limit for filament penetration. Percentage of invading cells from time-lapse experiments (4 independent experiments for each PDMS stiffness, Young’s modulus indicated, 40:1 and 30:1) was quantified (*n* = 50–60 cells per determination from a total of 900–1200 cells) using wide-field microscopy. Bars indicate SD and ****p < 0.0001. **b** Filament extension rate is linear during surface and invasive growth. Cells grown on or in PDMS at indicated stiffness (35:1 and 40:1) over 2 h, with images every 5 min. Filament length was determined from the sum projection GFP images and normalized to an initial length of 10–12 μm. **c** Filament extension rate is reduced upon invasive growth. The extension rate was determined from the time-lapse acquisition of cells grown within PDMS at indicated stiffness (35:1 and 40:1). Surface growth was carried out on PDMS with a stiffness 190-85 kPa (30:1–40:1). Quantification from 2 to 4 independent experiments, *n* = 20–30 cells per PDMS stiffness. Bars indicate SD with ****p < 0.0001 and *p < 0.05. **d** Invasively growing filaments do not undergo substantial changes in the *z*-position. Cells expressing plasma membrane GFP were imaged (green; middle panel) after growth in ~ 150 kPa PDMS (35:1), chambers that were labeled with Alexa-633 ConA (magenta; left panel), and 100 × 0.2 μm *z*-sections were acquired with DIC image shown (right panel). XY (upper panels) and XZ maximum projections are shown with dotted lines indicating the upper and lower limits of the microchamber and the green arrow indicating the center in the *z*-axis of the filament tip

**Fig. 8:**
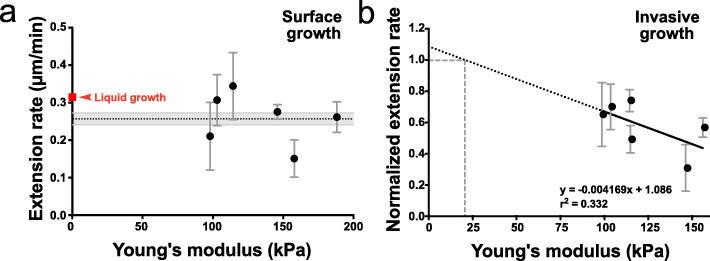
Filament extension rate on the PDMS surface is slightly reduced compared to that in liquid media. **a** Filament extension rate on the PDMS surface is independent of stiffness and reduced compared to that in liquid media. The extension rate was determined as in Fig. 7c. Filament lengths of cells grown in serum-containing liquid media (*n* = 75–80 cells) were measured every 30 min with mean and SEM shown in red. Surface extension rates determined as in Fig. 7c (*n* = 30 cells; 3–9 experiments per PDMS stiffness). Bars indicate SD. The mean of all surface extension rates (dotted black line) and SEM (gray zone) are indicated. **b** Cells experience a resistive force on the PDMS surface. Invasive extension rates determined as in Fig. 7c (*n* = 53 cells; 5–10 experiments per PDMS stiffness) and normalized to surface extension rates, with bars indicating SD and dotted line the best fit. At *Y* = 1, *X* is ~ 20 kPa

The buckling of the filaments, as well as the deformation of the PDMS wells during invasive growth, indicated that these filaments were responding to resistive force, whose magnitude we have measured in the physical model. The percentage of cells that penetrate PDMS is dependent on Young’s modulus (Fig. 2d, f), and analyses of percent of PDMS invasion at two stiffness values indicate that the threshold for invasion is between 120 and 200 kPa (Fig. 7a). Hence, to investigate the effects of resistive force on filamentous growth, we determined the length of the filaments over time from cells growing on and within PDMS in this range of stiffness. Figure 7b shows that cells extend at a constant rate, which is reduced by ~ 30% within PDMS from an average of 0.28 ± 0.07 μm/min (*n* = 29) for surface growth to 0.19 ± 0.05 μm/min (*n* = 32) when filaments were growing within PDMS with a stiffness of ~ 100 kPa (Fig. 7c). This filament extension rate was further reduced to 0.15 ± 0.08 μm/min (*n* = 23) upon growth in PDMS with a stiffness of ~ 150 kPa (Fig. 7c). To confirm that the reductions in extension rate during invasive growth were not due to the substantial changes in the *z*-position of the growing filament apex, cells were grown in PDMS chambers that had been stained with fluorescent ConA and *z*-section images were projected onto the XZ plane (Fig. 7d). These projections show that the filaments grow slightly downward in PDMS, below the bottom of the chamber, with maximally 5 μm displacement in the *z*-axis for a 25-μm filament, resulting in at most a 2% reduction in extension rate upon projection in the XY plane. In contrast, Fig. 8a shows that the mean filament extension rate of cells grown on the surface is not dependent on the substrate’s stiffness, with indistinguishable rates on PDMS with Young’s modulus from 100 to 200 kPa. Furthermore, the extension rate of invasive growth normalized for that of surface growth from each experiment correlates with substrate stiffness (Fig. 8b). Extrapolation to the *y*-intercept, where the invasive extension rate, equals the surface rate indicates a substrate stiffness of ~ 20 kPa, suggesting that during filamentous growth on PDMS, the cells experience this resistive force from adhesion. Consistently, the surface extension rate was slightly reduced compared to that in liquid media (0.26 ± 0.09 μm/min compared to 0.32 ± 0.01 μm/min; *p* = 0.001) (Fig. 8a). Of note, very few cells invaded PDMS at 200 kPa, on average ~ 5% (Fig. 7a), but, strikingly, the filament extension rate for these “escapers” was similar to that of less stiff PDMS, raising the attractive possibility that these cells may play a critical role in tissue invasion.

**Fig. 9:**
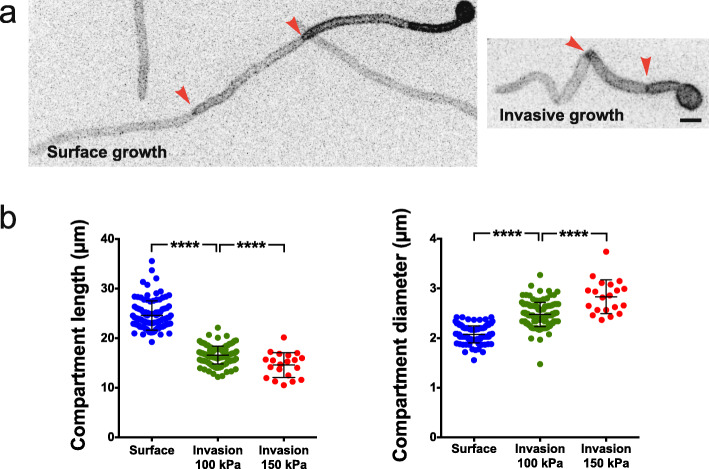
Filament morphology is altered during invasive growth. **a** Sum projection images of cells expressing GFP-Ct_Rac1_ grown in the presence of FCS on or within PDMS of 115 kPa stiffness, for 3h15 or 2h15, respectively. Arrowheads indicate the septa that delimit the measured compartment. **b** Compartment length is reduced and diameter is increased when cells are grown within a substrate of increasing stiffness (40:1 and 35:1). The length between two septa (left panel) along with the filament diameter (right panel), either the 2nd or the 3rd compartment from the tip, was measured in *n* = 20–115 cells, 2–22 experiments. SD are indicated and *p* < 0.0001

**Fig. 10:**
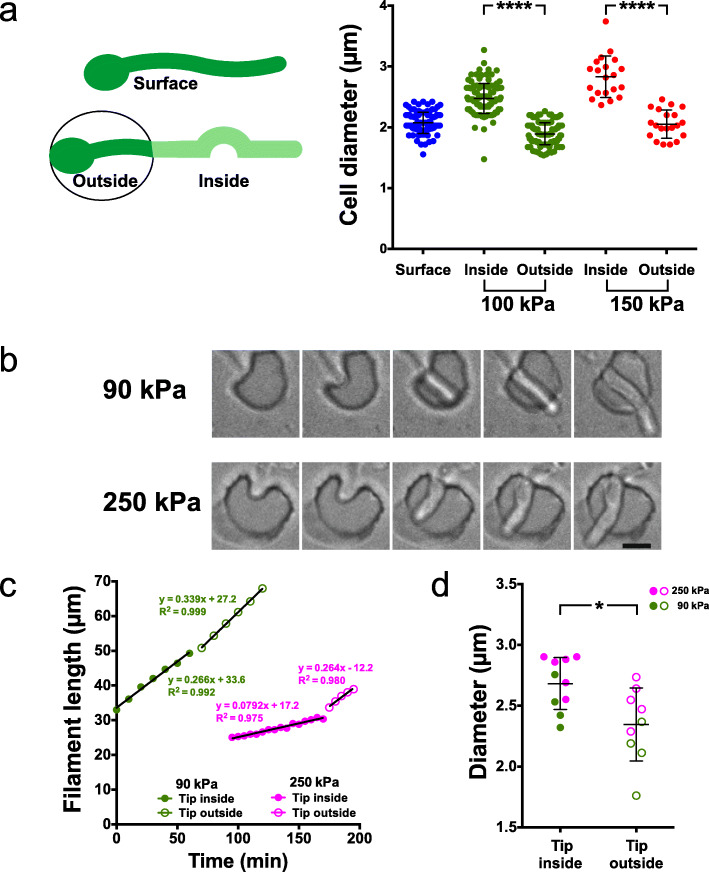
Filament diameter increases during growth in PDMS and decreases upon bursting out. **a** Filament diameter inside and outside PDMS at two stiffness values. The diameter of the filament was measured as in Fig. 9a and b (surface and portion inside PDMS). The diameter of the filament adjacent to the mother cell neck in the chamber was measured (portion outside indicated in the schematic). Bars indicate SD, with *p* < 0.0001 between inside and outside for each PDMS stiffness value (40:1 and 35:1). **b** Images of filament bursting out of PDMS. Cells grown in PDMS at indicated times over 2 h, with DIC images every 5 min (~ 250 kPa; 30:1) or 10 min (90 kPa; 40:1) shown. **c** Filament extension rate increases upon exiting PDMS. Filament length was measured from the sum projection GFP images prior to (green and magenta-filled symbols) and following emerging from PDMS (green and magenta-open symbols). **d** Filament tip diameter is slightly reduced upon emerging from PDMS. The tip diameter was determined at 4–5 times before or after exiting PDMS from **b** and **c**. Bars indicate SD and *p = 0.01

The difference in filament extension rate could either be due to an overall reduction in cell growth or a reduction in polarized, apical growth. To differentiate between these possibilities, we determined the length and diameter of compartments (between 2 septa) for cells growing on the surface and within PDMS (100 kPa). Figure 9a and b show that the compartment length decreased ~ 30%, from 24.6 ± 3.0 μm (*n* = 100) for surface growing cells to 16.6 ± 1.8 μm (*n* = 120) during invasive growth, and the filament diameter increased concomitantly from 2.1 ± 0.2 μm for surface growing cells to 2.5 ± 0.2 μm during invasive growth. As a result, the compartment volume remained constant (83 ± 17 μm^3^ for surface growing cells compared to 80 ± 18 μm^3^ for invasively growing cells), indicative of altered polarized growth. Consistently, analyses of filamentous cells grown within stiffer PDMS (150 kPa) revealed a further decrease in compartment length (14.6 ± 2.5 μm) and an increased diameter (2.8 ± 0.3 μm). This altered morphology is dependent on growth against a resistive force in PDMS as the diameter in the part of the filament outside PDMS was similar to that of surface growing cells (Fig. 10a). In addition to the comparison of cells growing on the surface and within PDMS, we examined the relatively rare occurrence of cells transitioning between these growth modes. Figure 10b and c show an example of such a transition, in which the extension rate is reduced during invasive growth in PDMS and increases exiting PDMS. Measurements of the filament diameter just before and after the filament exited PDMS revealed an increased filament diameter during invasive growth that was significantly reduced upon exiting PDMS (2.7 ± 0.2 μm compared to 2.3 ± 0.3 μm, Fig. 10d).

Reduced filament extension rate in response to a resistive force suggested that similar effects could be observed in cells undergoing non-invasive, dramatic subapical bending in chambers of stiff PDMS (Additional file 1: Figure S4A). Additional file 1: Figure S4B shows that, in such conditions, there was indeed a dramatic reduction in filament extension rate with an average (over 100–150 min) of 0.10 ± 0.01 μm/min. Surface growth rates were constant over time, whereas extension rates of cells undergoing dramatic subapical bending decreased concomitantly with the cell filling the well (Additional file 1: Figure S4C); initial rates of extension were 3-fold reduced from surface growth, and these were further reduced 3-fold after 2 h of growth. Due to the complex geometries during such a growth mode, we were unable to determine the resistive force that the filament experiences while it fills up the chamber; however, the initial extension rate is similar to that of filaments growing invasively in 150 kPa PDMS.

### Determination of the effective turgor pressure

From the comparison of extension rates within PDMS of different stiffness (Fig. 7c), we determined the effective turgor pressure in *C. albicans* hyphae, using the viscoplastic growth model [21]. This determination makes use of *F*_invas_ values, measured from the physical model (Fig. 6), after scaling these forces from a cylinder with a radius of 0.5 mm to that of 1.04 μm. In order to correctly extrapolate the macroscopic measurements to the microscopic scale of filamentous cells, we analyzed the physical forces at play. The mode of extension during hyphal growth and in this physical experimental model is different, as new material is incorporated into the hyphal tip, i.e. growth occurs via apical extension, whereas in the physical experimental model, the probe is pushed into the PDMS from the back. Given that only a small portion of the filamentous cell apex extends in the PDMS, we removed the contribution from friction/adhesion due to the displacement of a 1-mm-diameter probe within PDMS by subtracting the *F*_out_ value from the *F*_invas_ (Fig. 6c). These corrected *F*_invas_ values, i.e. *F*_in_, were largely independent of probe displacement rates over a range equivalent to cell filament extension rates when scaled down (0.2–0.4 μm/min). We used the equation that was established for *S. pombe* by Minc and colleagues;

1 $$ \frac{V_{(F)}}{V_o}=\left(1-\frac{F_{\left(\mathrm{PDMS}\right)}}{\pi {R}^2\Delta  P}\right) $$

*V*_(*F*)_ and *V*_*o*_ are the filament extension rates within PDMS and on the surface, respectively; *F*_(PDMS)_ is the resistive force of PDMS during filament displacement within this material (*F*_in_); *R* is the filament radius; and *∆P* is the effective turgor pressure. The *F*_(PDMS)_ was 1.5 ± 0.7 N and 0.7 ± 0.3 N at PDMS to cross-linker 35:1 (Young’s modulus of 150 kPa) and 40:1 (Young’s modulus of 100 kPa), respectively; scaling to the size of the hyphal filament (radius 1.04 μm for surface growth) yielded 6 ± 3 μN and 3.2 ± 1.4 μN, respectively. From these values, we determined the effective turgor pressure, *∆P*, to be 6 ± 3 MPa and 2 ± 1 MPa in these two conditions, respectively. Given that the hyphal filament diameter increases during invasion (radius 1.42 μm at Young’s modulus of 150 kPa and 1.24 μm at Young’s modulus of 100 kPa), the calculated *∆P* are 3.1 ± 1.5 MPa and 1.5 ± 0.7 MPa, suggesting that the hyphal turgor pressure is 1–3 MPa. This value for turgor pressure is within the range reported both for planktonic and biofilm *C. albicans* cells, ~ 1.2 MPa [19] and ~ 2 MPa [34], respectively, as well as *S. pombe*, 0.85–1.5 MPa [21, 35]. Nonetheless, it must be noted that these values are effective turgor pressure. In other words, *∆P* is the turgor pressure exceeding the critical stress needed to deform the cell wall [21]. Hence, a combination of local compartment turgor pressure alteration, difference in cell wall deformability, or potentially finer adjustments in tip geometry may play important roles in penetration and invasion.

### Resistive force affects cell polarity

**Fig. 11:**
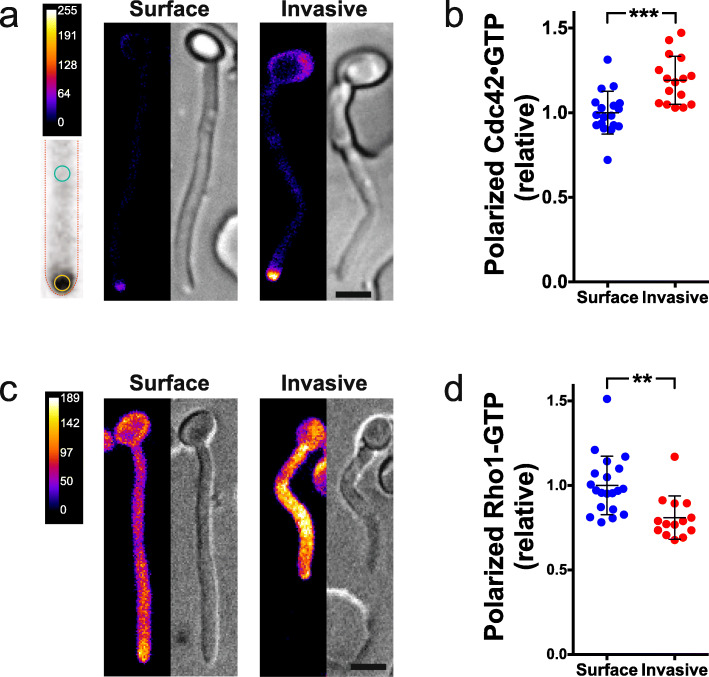
Invasively growing filaments have increased levels of active Cdc42 at the tip. **a**, **b** Tip-localized Cdc42•GTP is increased during invasive growth. **a** Representative sum projection and DIC images of cells expressing CRIB-GFP on or in 35:1 PDMS (~ 150 kPa). False colored sum projection of 23 × 0.4 μm *z*-sections (LUT, top) with schematic indicating regions quantitated at the tip and 5–10 μm back from the tip (bottom). **b** Mean Cdc42•GTP at the tip and 5–10 μm subapically determined from 4 independent time-lapse experiments (images every 5 min for ~ 2 h and sum projections of 23 × 0.4 μm *z*-sections; *n* = 16–18 cells). Polarized Cdc42•GTP is the tip signal divided by the subapical signal, 5–10 μm behind the tip (3.5-fold enrichment apically for surface growing cells, normalized to 1). Bars indicate SD and ***p = 0.0002. **c**, **d** Active Rho1 is delocalized during invasive growth. **c** Representative sum projections and DIC images as in **a** of cells expressing GFP-RID after growth on or in 35:1 PDMS (~ 150 kPa). **d** Mean Rho1•GTP at the tip and at 5–10 μm subapically was determined from 3 independent time-lapse experiments, as in **b** (*n* = 14–20 cells). Polarized Rho1•GTP is the tip signal divided by the subapical signal (1.4-fold enrichment apically for surface growing cells, normalized to 1, as in **b**). Bars indicate SD and **p = 0.001

The change in morphology during invasion, resulting in shorter and wider cells, could be explained by tip growth becoming more isotropic in response to a resistive force, raising the possibility that cell polarity is adversely affected. In *S. pombe*, it was observed that reducing the growth rate chemically, genetically, or mechanically destabilized active Cdc42 polarization, a cell polarity master regulator [36]. To investigate whether cell polarity was altered in hyphal filaments growing invasively in PDMS, we examined the distribution of active Cdc42 (Cdc42•GTP), using a CRIB-GFP reporter [37]. Surprisingly, we observed a striking increase in polarized active Cdc42 at the filament tip throughout invasive growth, compared to surface growth (Fig. 11a, b). We determined, using a tailor-made MATLAB program, that this results from an increase in the concentration of Cdc42•GTP at the tip, rather than an alteration in the position of the maximum signal or spread of active Cdc42 further down the filament (Additional file 1: Figure S5A-D). These results suggest that, in response to a resistive force, there is an increase in cell polarization, perhaps reflecting a direct response to such external forces. We speculate that this higher level of active Cdc42 during invasive growth is due to the increased recruitment of the Cdc42 activator, Cdc24 [38]. We next examined active Rho1, as cell wall stress mediated by the cell surface mechanosensors Wsc1/Mid2 results in Rho1 depolarization in *S. cerevisiae* [39, 40]. Figure 11c and d show that, in contrast to the increase in tip localized active Cdc42, active Rho1 is depolarized during invasive growth. We attribute this depolarization of active Rho1 to the mechanical properties of PDMS, which are likely to impose a uniform force over the hyphal filament surface, in addition to the resistive force in response to the tip extension.

**Fig. 12:**
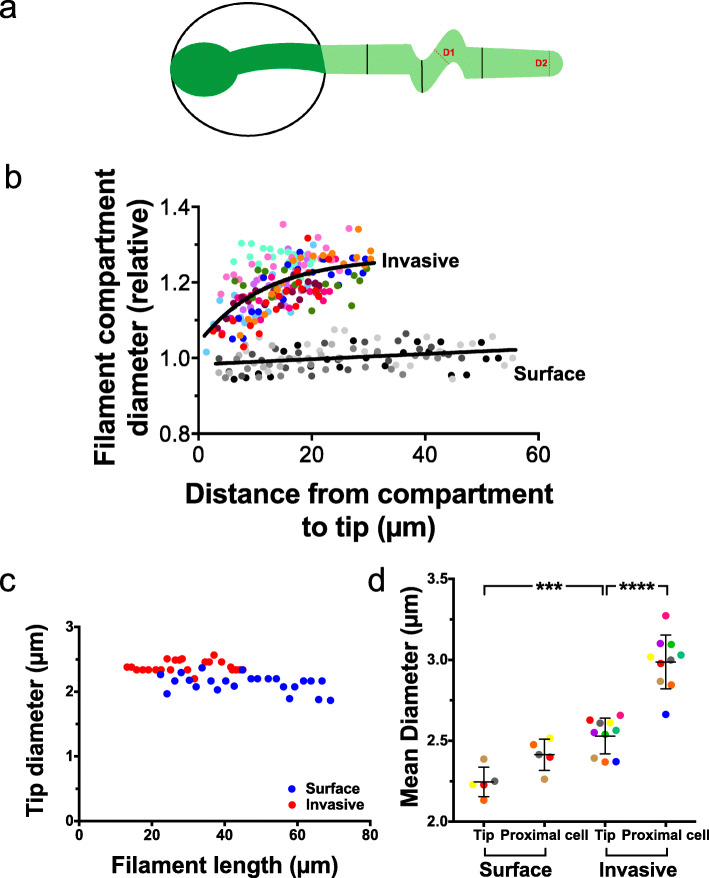
Mechanical forces are critical for filament morphology changes. **a** Schematic of filament during invasive growth. Lighter green indicates a portion of the filament within PDMS; D1, compartment or proximal cell diameter; and D2, tip diameter. **b** The diameter of the cell compartment increases as it becomes further away from the filament tip during invasive growth. Cell compartment diameter (measured at 5 equidistant positions between 2 septa), initially on average 5 μm from tip, was determined from 3 independent experiments acquired as in Fig. 11b (*n* = 5–10 cells) grown on or within 35:1 PDMS (~ 150 kPa). Distance from the compartment center to the tip was determined at each time. The diameter was normalized to the diameter of the last time point for each cell and further normalized by the mean difference between the final compartment diameters of invasive cells compared to surface cells. Each color represents a cell. **c** Filament tip diameter does not vary as a function of filament length for surface and invasive growth. Tip diameter (2 μm back from the apex) measured for one cell growing on PDMS surface and one cell growing within PDMS, over a 2-h time course. **d** Tip diameter increase does not underlie proximal cell diameter increase during invasive growth. Tip and proximal cell diameters were measured from the same cells as in **a**. Proximal cell diameter was measured at the center of the cell proximal to the apical cell over the 2nd hour of the time course (filaments > 30 μm long). Bars indicate SD, with ****p < 0.0001 between the tip and the proximal cell during invasive growth and ***p = 0.0003 between the invasive and surface tips

The increase in tip-localized active Cdc42 during invasive growth suggests that the increase in filament diameter does not result from growth becoming more isotropic in response to resistive force. To examine whether this morphological change results from mechanical forces, we compared cell morphology over time during growth on the surface and within PDMS. Figure 12a and b show that the relative filament diameter (D1) was not altered during surface growth (mean diameter 2.26 ± 0.15 μm initially compared to 2.44 ± 0.06 μm after 2 h growth), in contrast to the invasive growth where there was a striking increase (2.38 ± 0.22 μm initially compared to 2.98 ± 0.15 μm, *p* < 0.0001). Specifically, the diameter of the filament compartment increased even > 10 μm back from the tip (Fig. 12b). This ~ 25% increase in diameter during invasive growth could either occur upon tip growth or subsequent to tip growth. The hyphal tip diameter was constant over 2 h of invasive growth and only slightly wider than that of cells growing on the surface (2.53 ± 0.11 μm compared to 2.24 ± 0.09 μm; *p* = 0.0003) (Fig. 12c, d). In contrast, Fig. 12d shows that there was a significant difference between the mean diameter at the tip of the apical cell and that of the cell proximal to the apical cell during invasive growth (mean proximal cell diameter 2.99 ± 0.17 μm; *p* < 0.0001). Together, these results indicate that relatively small changes in the tip morphology are not sufficient to explain the altered morphology of the filament, back from the tip, which are due to external mechanical forces.

## Discussion

We used PDMS micro-fabrication to probe the relationship between substrate stiffness and growth of *C. albicans* filamentous cells. Below a stiffness threshold of ~ 200 kPa, *C. albicans* can penetrate and grow within PDMS. The chemical inertness of this polymer, as well as the observed well deformation, suggest that turgor pressure-driven active penetration is critical for this invasive growth. *C. albicans* filamentous growth within a stiff substrate is characterized by dramatic filament buckling, which correlates with the position of cell division sites, in addition to a stiffness-dependent decrease in extension rate. Growth within a substrate also resulted in a striking alteration in morphology, i.e. reduced cell compartment length and increased diameter. Our results reveal that changes in morphology are not due to a depolarization of active Cdc42, but rather to the mechanical forces from the substrate.

### Growth behavior as a function of substrate stiffness

Substrate stiffness determines whether *C. albicans* will grow on a surface or within it, below a threshold corresponding to a stiffness of 200 kPa. During growth within PDMS, a substantial fraction of filaments buckle, with greater than 50% buckling in a substrate with a Young’s modulus of ~ 100 kPa. Cells trapped in stiff (200 kPa or greater) microchambers undergo dramatic subapical bending. While both filament buckling and subapical bending depend on filament extension, the former occurs at least 5–10 μm away from the apex whereas the latter occurs at the filament tip. In a number of cases, filaments buckled as the tip (within PDMS) approached a well, followed by partial release upon penetration into this well. This bursting out event is similar, in some respects, to damaging of or escaping from host cells, such as macrophages [28–31].

Despite the estimated resistive force during invasive growth of 3–10 μN at the hyphal scale, we did not observe dramatic changes in the filament tip shape. Interestingly, the location of cell division sites appeared to be affected by filament buckling, in that there was a high correlation between the division site location and the site of buckling. Septins have been shown to be enriched preferentially at locations of high curvature in *Ashbya gossypii* hyphal filaments, i.e. at branch points [41]. An attractive possibility is that the location of the cell division site at the buckle, or vice versa, may minimize physical stress or damage to the filament.

### Effects of substrate stiffness on invasive growth

Filament extension rate is reduced, and cell compartment length decreases with increasing substrate stiffness; however, cell volume is largely unaffected during growth within 100 kPa PDMS, due to an increase in filament diameter. These results suggest that the overall growth rate is unaffected in this condition, raising the possibility that the cell tip growth area is altered by resistive force. Given that altering growth in *S. pombe* by chemical, genetic, or mechanical means, results in destabilization of the active Cdc42 cluster at the growth site, we investigated the distribution of this key GTPase during invasive growth. Surprisingly, the distribution of active Cdc42 was not altered during invasive growth, but rather there was an increase in the concentration of active Cdc42 at the tip. In contrast, active Rho1, which is critical for glucan synthesis, was less polarized and observed throughout the filament during invasive growth. Together, these results suggest that, during invasive growth, cells sense resistive force from the PDMS and polarization increases at the tip, while remodeling their cell walls throughout the filament to counter this resistive force and/or the increase in turgor pressure. A likely possibility is that this resistive force is sensed by the mechano-sensing pathway, via a module composed of the mucin Msb2 and the cell wall protein Sho1 [42] critical for *S. cerevisiae* survival during compressive stress. In this yeast, Msb2 localizes to sites of growth and binds active Cdc42 [43], and cell wall stress has been shown to depolarize Rho1, as well as to hyperactivate this GTPase [39, 40] via the cell surface sensors, Wsc1 and Mid2. Indeed, *S. cerevisiae* cells sense compressive stress via Mid2, which localizes uniformly at the plasma membrane [44]. We speculate that the increased compression around the hyphal filament leads to depolarized active Rho1 via Mid2, in contrast to localized active Cdc42 via Msb2.

We have used a viscoplastic model for fungal growth that was originally established to describe cell shape control in plants [45, 46] and used, more recently, to analyze cell growth in fission yeast [21]. In such models, viscoplastic deformation of the cell wall underlies growth, which is driven by high turgor pressure. Specifically, the pressure (*P*) has to exceed the threshold plastic yield strain (*P*_*c*_) of the cell wall—the cell growth rate is proportional to the wall strain that exceeds this threshold: ${V}_o\propto \frac{\left(P-{P}_c\right)}{E_{\mathrm{CW}}}$. Hence, we have used Eq. (1) to derive the effective turgor pressure in *C. albicans* hyphae. Here, we used the differences in growth rates on PDMS surface and within PDMS, as well as an approximation of the external force. This latter force was calculated based on the displacement within PDMS of a 1-mm-diameter steel probe, minus the contribution from friction/adhesion and scaled to the hyphal diameter, assuming scaling with respect to the cross-sectional area and the absence of dramatic changes in geometry. From this analysis, we determined the effective turgor pressure (*∆P* = *P* − *P*_*c*_) to be 1.5 and 3.1 MPa, in 100 kPa and 150 kPa PDMS, respectively. Either increasing filament diameter or decreasing extension rate results in a lower ∆*P*. The physical experimental model, however, does not take into account the geometry of the hyphal tip, which has a different curvature than the metal probe, which could result in an overestimation of the forces in the physical model. More precise measurements of forces relevant to hyphae will require a more accurate description of the hyphal tip size and geometry. One possibility is that, upon growth in stiffer PDMS, there is an increase in turgor pressure; our results are consistent with such a scenario as *∆P* increases from 1.5 to 3.1 MPa, with an increase in PDMS stiffness from 100 to 150 kPa. This increase in turgor pressure could be responsible for the increased volume of the invasive cell compartment within 150 kPa PDMS (92 μm^3^ compared to 83 μm^3^ for surface growing cells, *p* = 0.045). Previous studies on cell wall expansion in *S. pombe* [35] have shown that $\frac{\Delta  P}{Y}=\frac{\left({R}^{\ast}\right)t}{R_1}$; where *Y* is the cell wall Young’s modulus; R^*^ is the expansion ratio in width ([*R*_1_ – *R*_0_]/*R*_0_); *R*_1_ and *R*_0_ are the cell radius within the PDMS and on the surface, respectively; and *t* is the cell wall thickness (~ 0.2 μm). Assuming *Y* is constant at these two PDMS stiffness conditions, $\frac{\Delta  P}{Y}\left(35:1\right)$ is 1.7-fold greater than $\frac{\Delta  P}{Y}\left(40:1\right)$, consistent with the observed increase in turgor pressure we derived from the growth rate difference. The slightly reduced extension rate on PDMS surface compared to that in liquid, together with the extrapolation of normalized extension rate as a function of PDMS stiffness to where $\frac{V_{(F)}}{V_o}=1$, indicates that during growth on a surface, the hyphae experience a small resistive force, equivalent to growth within less stiff PDMS (~ 20 kPa), which we attribute to adherence/friction. It is likely that depending on the cellular surface, the contribution of adherence/friction will vary.

### Cell morphology is dependent of substrate stiffness

Our results indicate that effects on cell morphology are only observed in filaments within the PDMS, suggesting that resistive forces from the PDMS lead to an alteration in morphology. Strikingly, during growth in the stiffest PDMS, we observed a progressive increase in compartment diameter, even when this compartment was more than 10 μm back from the hyphal tip. This could be due to the additional modification of the cell wall in this proximal compartment, i.e. resulting in a less stiff cell wall, an increase in turgor pressure, and/or mechanical deformation of the filament. Although we cannot rule out that the cell wall in this proximal compartment is less stiff during invasive growth, we favor the latter two possibilities, as the cell compartment volume increases and ~ 60% of invasively growing hyphae buckle in this PDMS stiffness (150 kPa). The dramatic alteration in filament morphology was not solely due to a widened tip, as it only increased slightly during invasive growth, compared to the proximal compartment that increased 25% more than the tip diameter. It is likely that these morphology changes are due to the mechanical forces from growing against a resistive substrate together with an increase in turgor pressure.

A number of fungal pathogens penetrate host tissues, including medically relevant *C. albicans* [8, 10, 12] and *A. fumigatus* [9], and plant pathogens [47, 48], including *Colletotrichum* sp. [49], *Ustilago maydis* [50], *Magnaporthe* sp. [51, 52], and *Fusarium* sp. [53]. For the latter plant pathogens, high turgor pressure is generated inside a specialized cell, called an appressorium, that generates pressures in excess of 8 MPa [51]. Blocking host cell endocytosis of the human fungal pathogens *C. albicans* [8, 15–17] and *A. fumigatus* [9] with Cytochalasin D has revealed that both of these fungi can enter epithelial tissue by active penetration. Young’s moduli for mammalian host cells are in the 1–100 kPa range. Hence, the effects we observe on filament extension rate and morphology are likely to be relevant during active penetration of epithelial cells, and it is attractive to speculate that turgor pressure, in particular the osmolytes critical for generating this force, might be a target for antifungal drugs.

## Conclusions

Our results suggest that the stiffness of host cells dictates which cell type *C. albicans* can penetrate. Interestingly, even with stiffer PDMS (~ 200 kPa), we observed a small percentage of cells that were able to invade the substrate, suggesting these cells have specific properties that could be an advantage during epithelium invasion.

## Methods

### Strains, media, and genetic methods

Standard methods were used for *C. albicans* cell culture, molecular, and genetic manipulations as described. Derivatives of the BWP17 strain were used in this study and are listed in Table S1. Strains were grown in rich media (yeast extract peptone dextrose) at 30 °C for all experiments, and induction of filamentous growth was carried out with fetal calf serum (FCS) at 37 °C. Oligonucleotides and synthesized DNA used in this study are listed in Tables S2 and S3. pDUP5-mScarlet-Ct_Rac1_ was generated by PCR amplification of CamScarlet with a unique 5′ AscI site and a 3′ CtRac1 followed by a unique MluI site (oligonucleotides CamScarletAscIp and yemChmCtRacMluI) and cloned into pDUP5-ADH1p-CibN-Ct_Rac1_-ACT1t [33] resulting in pDUP5-ADH1p-CamScarlet-Ct_Rac1_-ACT1t. The nucleotide sequence for RFP mScarlet [54] was codon optimized for *C. albicans* and commercially synthesized (Genscript). mScarlet was PCR amplified with unique PstI and AscI sites (oligonucleotides GA3CamScarPstIp and CamScarletmAscI) and cloned into a pFA-GFPγ-URA3 backbone [55] resulting in pFA-CamScarlet-URA3. The RID, CRIB, and Ct_Rac1_ plasmids were linearized with StuI or NgoMIV and transformed into strains.

### Micro-fabrication

Microchambers were fabricated using standard soft-lithography methods [56, 57]). Chambers were 5 μm deep and 10 μm in diameter with 15 μm spacing between them. The overall thickness of microchamber preparations was approximately 150 μm. PDMS microchambers of varying stiffness were generated by varying the ratios of polymer and cross-linker (Sylgard 184; Dow Corning). Polymer-cross-linker mixtures were spin coated (Laurell Technologies corporation) on molds (5 s 100 rpm followed by 50 s 500 rpm) to achieve ~ 150 μm thickness and then cured for 1 h at 60 °C PDMS (10:1). Thick PDMS frames were then placed on top of the partially cured chambers, which were baked for an additional 2 h at 90 °C, in order to peel the thin chambers off the mold. For viscoanalyzer measurements, 20 × 20 × 5 mm PDMS pieces were similarly cured and samples subjected to an amplitude of oscillation of 5–150 μm at 10 Hz frequency using a DMA3007000 Metravib 52 (Areva) with Dynaset20 software. Young’s moduli were calculated using the following formula, $E=\frac{\sigma }{\varepsilon }=\frac{F\times h}{D\times l\times e}$; with *F* being the force, *D* the deformation amplitude, *H* the sample height, *e* the sample thickness, and *l* the sample length. For analysis of each PDMS preparation, measurements at 5 different deformation values (5–150 μm) were carried out, and the mean of 3 of these measurements was used for Young’s modulus determination. For indentation experiments, varying ratios of polymer and cross-linker were poured into two different sized cylindrical aluminum molds, 3.5 (*d*) × 1.75 cm (*h*) and 8 (*d*) × 2 cm (*h*), and cured as described above.

### Physical model of penetration

Half of the PDMS cylinders were adhered to an aluminum plate with a 5-mm-diameter hole. For the other half of the PDMS cylinders, the bottom of the mold was removed such that they were fixed around their circumference. Cylindrical PDMS pieces from both conditions were indented in their center with a hemi-spherical 1-mm-diameter steel probe, which was highly polished. This probe tip was machined with a slight conical shape ending in a spherical cap to approximate a hyphal filament tip and had a 2-daN force sensor (Kistler), as well as a displacement sensor (Fastar Data Instruments) attached. This probe was extended using a moving deck with a motor (Cerclet) at speeds of 1.6–3.2 μm/s. Measurements were made on average 10 samples at each PDMS ratio. From force versus displacement curves, the following forces were derived: the maximum value was the *F*_crit_, the mean of the first plateau following penetration *F*_invas_, and the mean of the second plateau (following the exit of the PDMS) *F*_out_. Forces were then averaged and scaled to the cross-sectional area of the hyphal filament. We assumed that in the physical model of penetration and in the *C. albicans* growth experiments, the PDMS behaved as a homogenous material, and hence, scaling in terms of size was carried out with a constant critical stress. This assumption is justified, as the diameter of the filament, ~ 2 μm, is 10^3^-fold greater than the PDMS mesh size, which is 2–3 nm with 40:1 PDMS. Hence, scaling of the force, as stress during rupture is constant, was achieved by dividing for the ratio of the metal probe/filament radii squared.

### Microscopy sample preparation

PDMS microchambers were activated by plasma treatment (Harrick Plasma Cleaner) for 14 s at 500 mTorr on low setting submerged in DH_2_O until usage. Prior to usage, PDMS samples were dried with nitrogen gas, treated with poly-d-lysine (1 mg/ml) and subsequently treated with concanavalin A (0.4 mg/ml), each incubated for 20 min followed by drying with a stream of nitrogen gas. Exponentially growing cells were mixed with FCS media (75% FCS, 0.6 × minimal media and 2% dextrose) and spotted onto the microchambers and were sealed with a coverslip. Typically, cells on PDMS microchambers were incubated for ~ 1 h at 37 °C prior to microscopy to initiate filamentation.

### Microscopy and image analysis

Cells were imaged as described [33] using either spinning disk confocal microscopy or wide-field fluorescence microscopy with a UPLANAPO 1.2 NA × 60 or Plan-Neofluar 0.75 NA × 40 objectives, respectively. Images were acquired at indicated times, with 0.4 or 0.5 μm *z*-sections (13 to 24) to capture the entire filament. For growth depth experiments, 100 × 0.2 μm *z*-sections were acquired. For the analyses of extension rate, compartment morphology, tip morphology, and cell depth images were deconvolved with the Huygens Professional software version 18.04 (Scientific-Volume Imaging) with recommended settings and a signal to noise ratio of 10, and sum projections were used. Reflection images in XZ were acquired on an upright Leica DM5500 TCS SPE laser-scanning microscope (Leica Microsystems, Mannheim, Germany) equipped with a galvanometric stage, using an APO 0.3 NA × 10 objective. Reflection images were acquired in XZ reflection mode using a 488-nm laser and an 80/20 dichroic filter. Scale bars, unless otherwise indicated, are 5 μm.

Image analysis for extension rate, compartment/tip diameter, and active Rho GTPase polarization was carried out with Fiji (version 1.51) [58]. For the determination of the extension rate, the filament lengths (mother cell neck to filament tip) were determined over time using plasma membrane fluorescence signal and values are from a curve fit of minimally 1 h acquisition (> 12 time points). For liquid extension rates, cells induced with 50% FCS at 37 °C were fixed every 30 min and average cell lengths for each time point (0–90 min) were used. Compartment lengths were measured using the edge-to-edge fluorescence signal between two formed septa. Only invasive compartments that were entirely within the PDMS were used for analyses. Active Cdc42 and Rho1 polarization was determined using a fixed size ROI to quantitate the signal intensity at the tip and in a subapical region. The ratio of the tip to subapical signal was averaged over the time-lapse, and the mean for invasive cells was normalized to the mean ratio for non-invasive cells. The percentage of cells undergoing subapical bending within a chamber and filament buckling within PDMS was assessed using DIC images. For the determination of errors from values derived from multiple measurements, classical error propagation was carried out [59]. Statistical significance was determined with Student’s *t* test.

For the representation of 3D DIC images, an algorithm was applied to image *z*-stacks using a custom-developed MATLAB program called InFocus inspired from Extended Depth of Field plugin [60]. The program extracts from any 3D multi-channel acquisition, the local contrast per channel. From these maps, it determines the *Z* of highest contrast per pixel from one or a combination of channels and then extracts a smoothened plane to get a 2D multichannel image. Parameters can be adapted through the graphical user interface. For quantitation of filament tip curvature over time, we developed another MATLAB program called TipCurve with an intuitive interface dedicated for morphological analyses along the major axis of filamentous cells. For such analyses, 3D images were converted into 2D images by sum projection, and similar to the HyphalPolarity program [33], a backbone was extracted from images over time, but also the cell contour, specifically extracting the tip first, we estimate the extremity of the backbone subtracted by the tip radius. Then, we estimate the local curvature only along the tip (either − 45° to + 45° or − 90 to 90°) from the curvilinear function f(s) extracted from cell contour:

$$ C(s)=\frac{1}{R(s)}=\frac{f^{{\prime\prime} }(s)}{{\left(f\prime (s)\right)}^2} $$

The distribution of active Cdc42 and Rho1 at the filament tip was determined by TipCurve. For these analyses, images that had a polarized fluorescent signal along with a uniform cytoplasmic signal were used. The latter was used to identify the cell and extract the backbone. Then, an additive projection on the backbone was done to get kymograph curves per time point and per fluorescence channel. To estimate the distribution of intensity at the tip of Cdc42 and Rho1, we fitted the kymograph to estimate *x*_*max*_, the distance of maximum of intensity from the tip and the decay of intensity above *x*_*max*_ as an exponential function. Both Infocus and TipCurve can be provided as executables on demand.

## Supplementary information

**Additional file 1: Figure S1.** Strain versus stress dependence of PDMS. Analyses carried out using a Viscoanalyzer, with oscillation at 10 Hz of PDMS at cross-linker ratio of 40:1. **Figure S2.** Invasive growth and penetration into adjacent chamber in PDMS of different stiffness. DIC time-lapse experiments at indicated PDMS:cross-linker ratio and measured stiffness (Young’s modulus). The adjacent chamber is highlighted with a dotted yellow line and deformation of this chamber lasted ~40 min with 40:1 PDMS ratio and 80-90 min for the two stiffer PDMS substrates. **Figure S3.** The shape of filament tip is not substantially altered during invasive growth in PDMS. A) Radius of curvature over time is constant in surface and invasively growing cells. Radius of curvature with an arc of ± 90° or ± 45° at the filament tip. B) Shape of filament tip of surface growing cells over time. Cells were grown on PDMS (30:1; 250 kPa) and 31 × 5 min GFP sum projections were analyzed. Radius of curvature with ± 45° by indicated open lines and ± 90° indicated by solid lines. **Figure S4.** Cells confined within a stiff PDMS chamber have reduced filament extension rates. A) Constricted growth within a PDMS chamber. Typical time-lapse experiment using 160 kPa PDMS, with DIC images every 5 min shown. B) Filament extension rate within a stiff chamber is not linear. Filament length was determined from images every 5 min for ~ 2 h and GFP sum projections (*n* = 9 cells). C) Filament extension rate is substantially reduced as chamber fills up. Initial (filament length 10-20 μm) and final (filament length > 20 μm) extension rates were determined from fits to 6 × 5 min GFP sum projections. (colors represent individual cells). Bars indicate SD and **** *p* < 0.0001. **Figure S5.** Distribution of active Cdc42 is not altered during invasive growth. A) Schematic indicating fluorescence signal over the filament long axis. Quantitation of slope of Gaussian farthest from tip in red (Max Slope, in relative units), distance maximum signal to tip (x_max_ in μm), and half width half max of the Gaussian farthest from tip in red (x_Spread_-x_max_), i.e. the signal spread (Spread in μm). Signal is denoted by I and distance from tip by x. B) Distribution of active Cdc42 during surface and invasive filamentous growth. Experiment described in Figure 11a and 11b with the mean signal for each cell (colors represents individual cells), normalized to the mean signal for tip Cdc42•GTP in surface growing cells. Bars indicate SD. C) Distribution of active Cdc42 is not altered upon invasive growth. Relative maximum slope (left), distance from maximum signal to the tip (middle) and spread of signal (right) determined from 6-8 cells, using tailor-made Matlab program. Bars indicate SD; surface and invasive cells were not significantly different. D) Apical and subapical active Cdc42 signals are stable over time. Relative signals from apical and subapical region of sum projections, normalized to maximum invasive subapical signal.

**Additional file 2:** **Movie S1.** Invasive growth and penetration into adjacent chamber. Cells grown with indicated stiffness PDMS and followed over time either by DIC optics or fluorescence of labeled with plasma membrane GFP.

**Additional file 3: Movie S2.** Invasively growing filaments have increased levels of active Cdc42 at the tip. False colored sum projections of cells expressing CRIB-GFP reporter for active Cdc42.

**Additional file 4: Table S1.** Strains used in the study [61, 62]. **Table S2.** Oligonucleotides used in the study. **Table S3.** Synthesized DNA used in the study.

## Data Availability

All the data on which the conclusions of the paper are based are presented in the paper and its additional files.
