# Supplementary figures and images for: Mechanical force-induced morphology changes in a human fungal pathogen
Source: BMC Biol. 2020 Sep 11;18:122. doi: 10.1186/s12915-020-00833-0 (PMC7488538; doi:10.1186/s12915-020-00833-0)

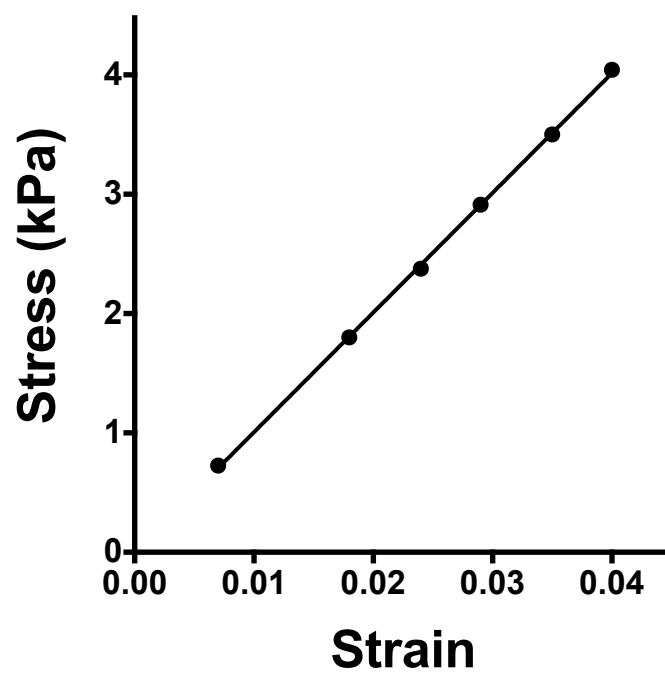

**Figure S1**

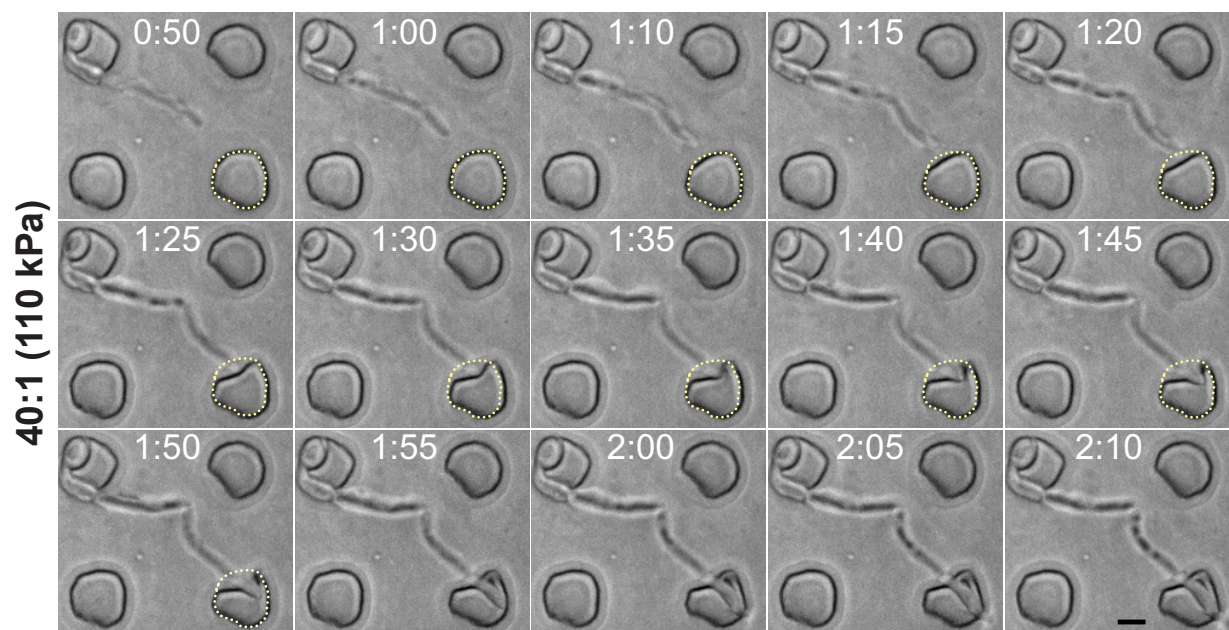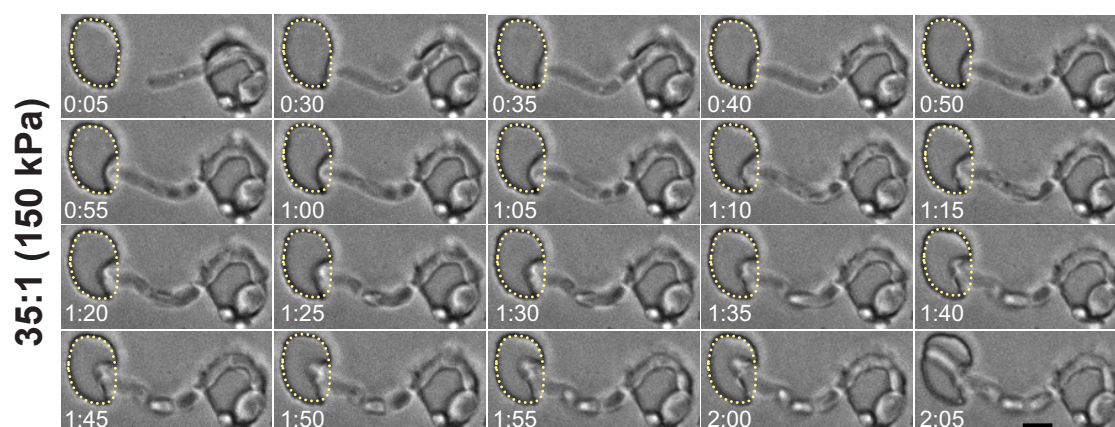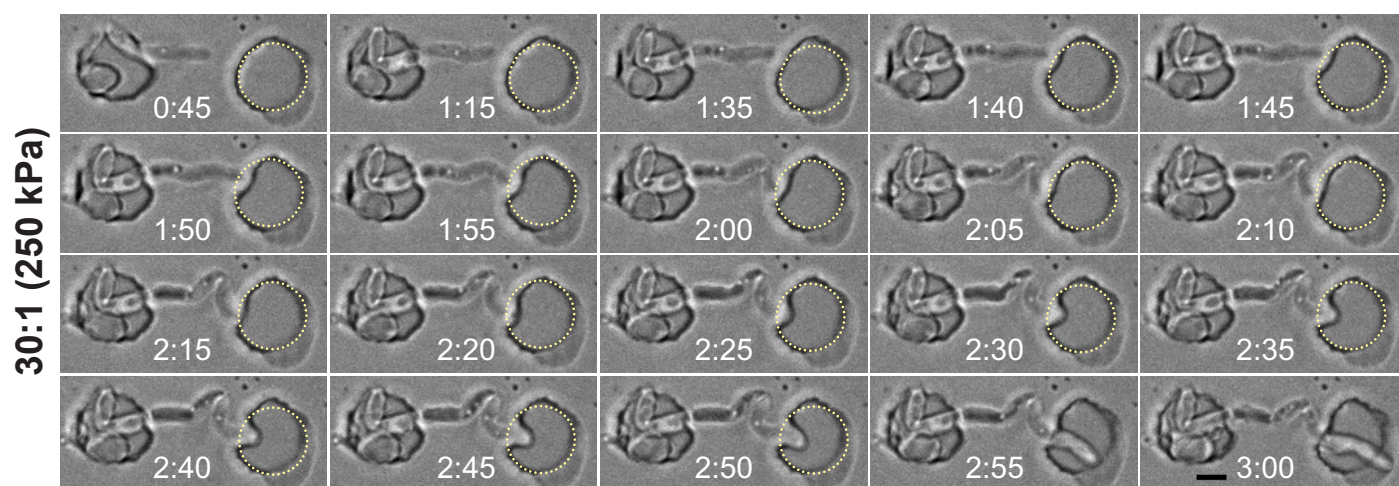

**Figure S2**

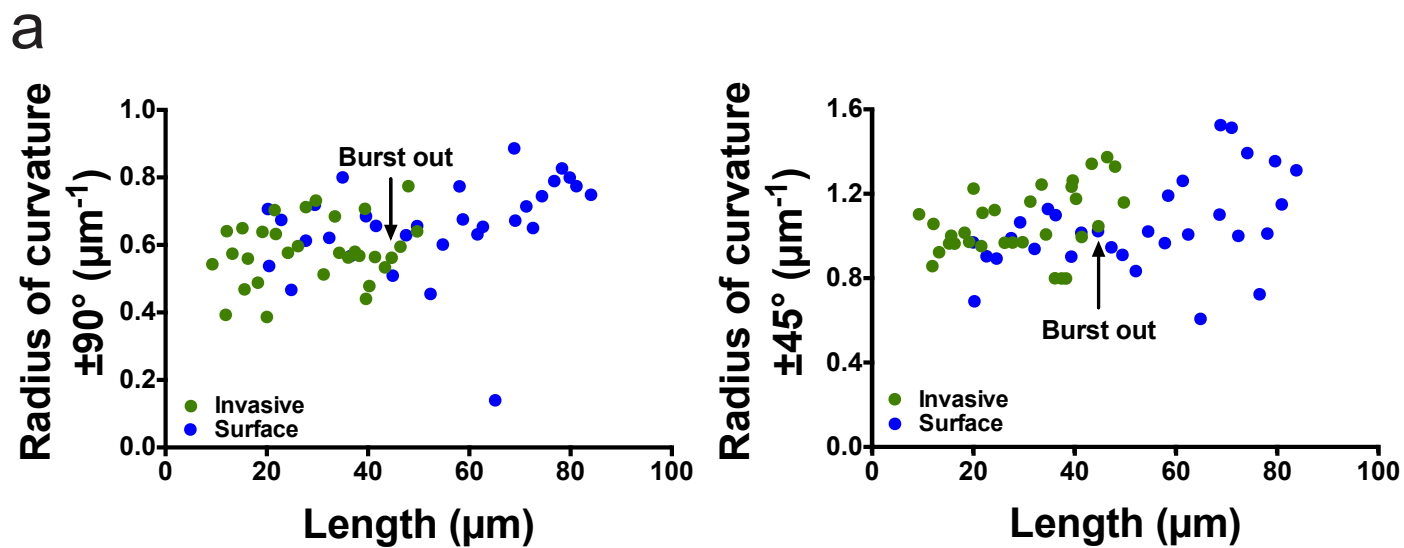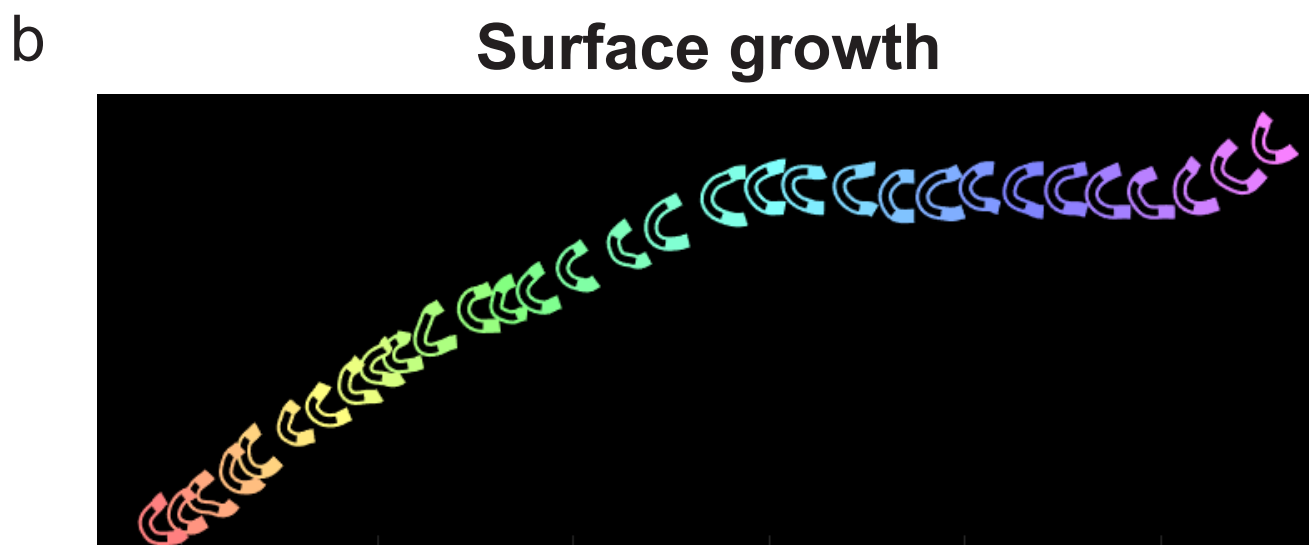

Figure S3

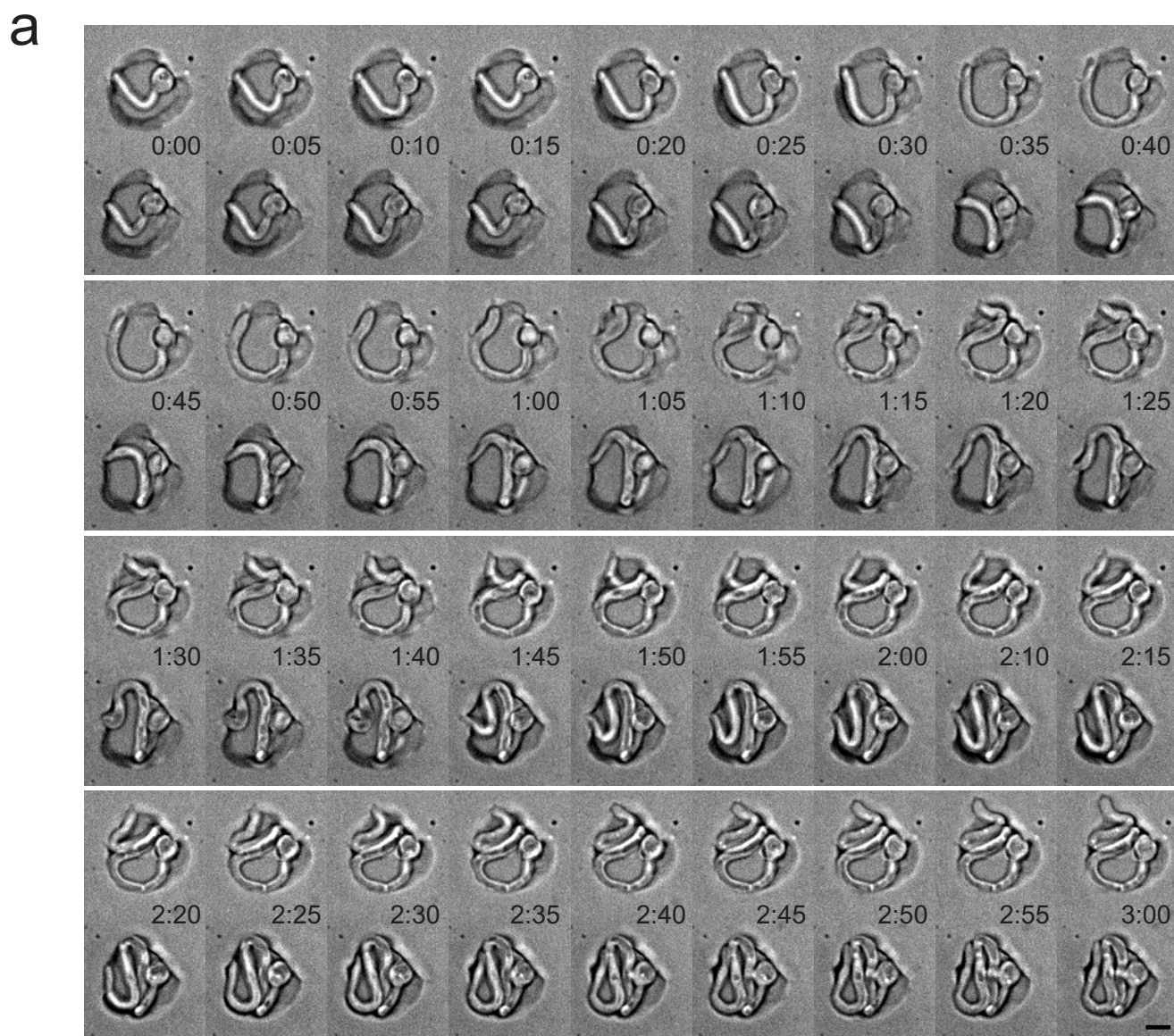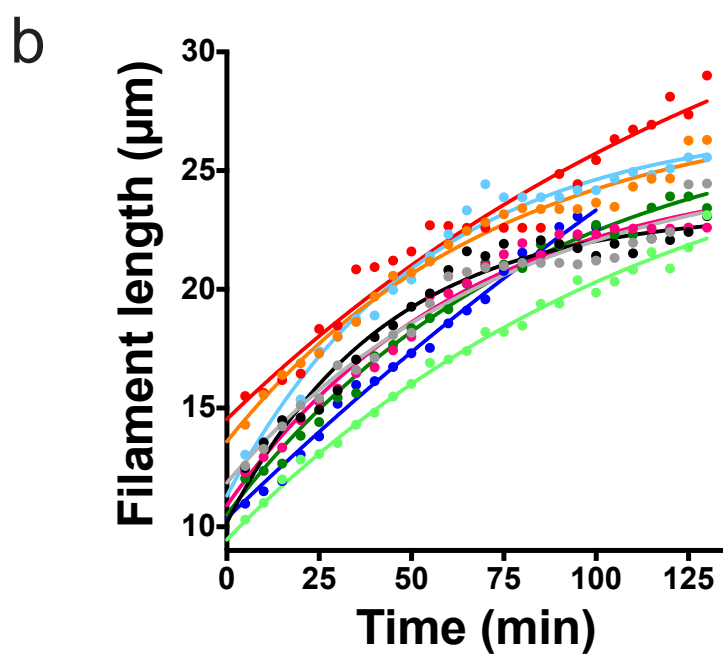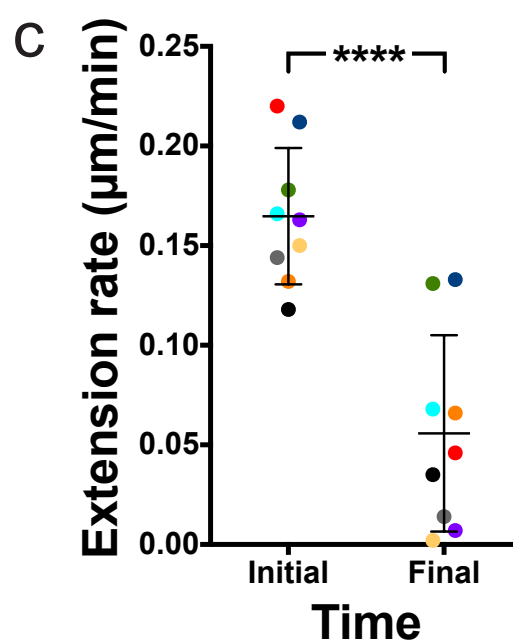

**Figure S4**

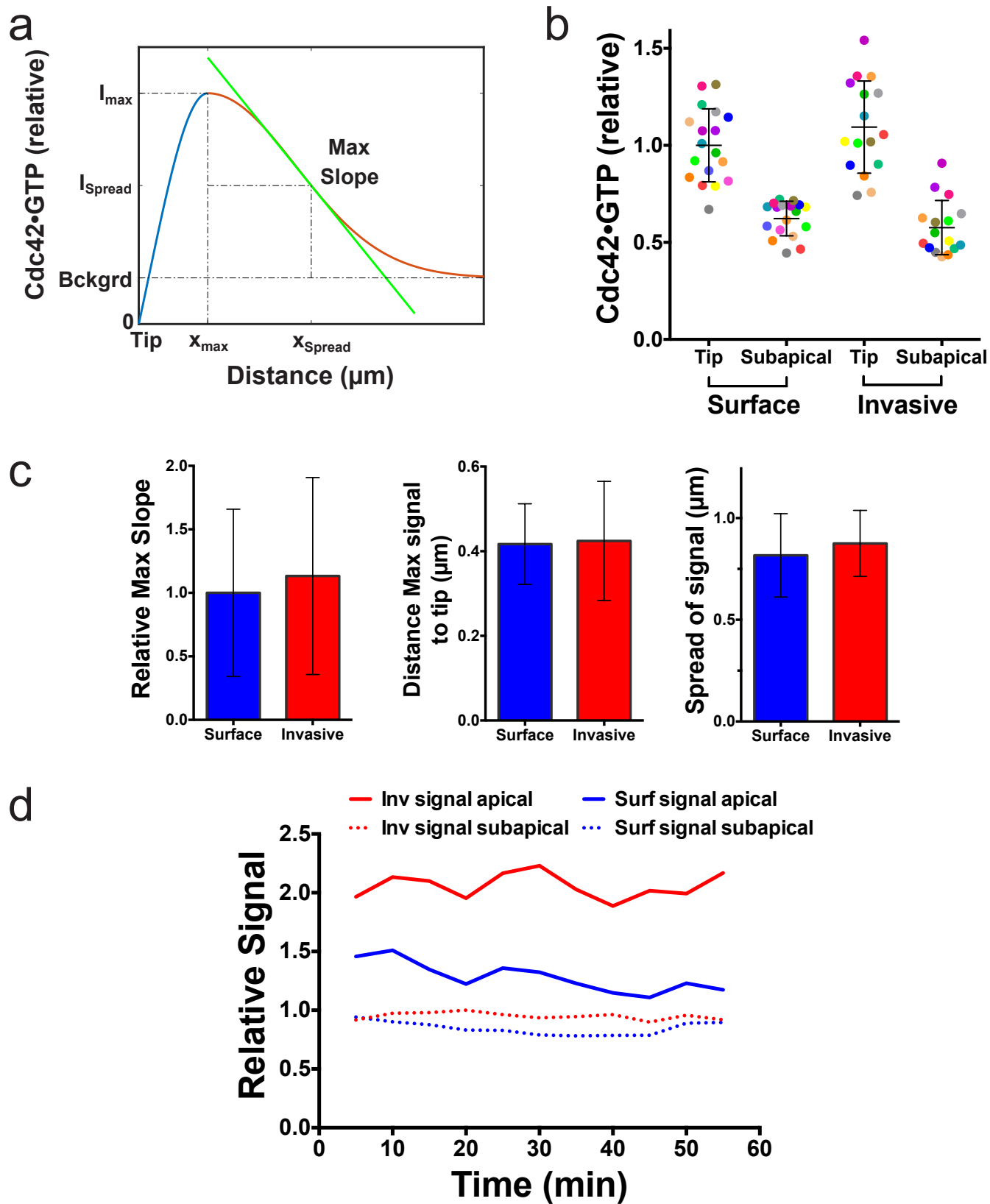

**Figure S5**

Supplement: Supplementary file 1 — Additional file 1: Figure S1. Strain versus stress dependence of PDMS. Analyses carried out using a Viscoanalyzer, with oscillation at 10 Hz of PDMS at cross-linker ratio of 40:1. Figure S2. Invasive growth and penetration into adjacent chamber in PDMS of different stiffness. DIC time-lapse experiments at indicated PDMS:cross-linker ratio and measured stiffness (Young’s modulus). The adjacent chamber is highlighted with a dotted yellow line and deformation of this chamber lasted ~40 min with 40:1 PDMS ratio and 80-90 min for the two stiffer PDMS substrates. Figure S3. The shape of filament tip is not substantially altered during invasive growth in PDMS. A) Radius of curvature over time is constant in surface and invasively growing cells. Radius of curvature with an arc of ± 90° or ± 45° at the filament tip. B) Shape of filament tip of surface growing cells over time. Cells were grown on PDMS (30:1; 250 kPa) and 31 × 5 min GFP sum projections were analyzed. Radius of curvature with ± 45° by indicated open lines and ± 90° indicated by solid lines. Figure S4. Cells confined within a stiff PDMS chamber have reduced filament extension rates. A) Constricted growth within a PDMS chamber. Typical time-lapse experiment using 160 kPa PDMS, with DIC images every 5 min shown. B) Filament extension rate within a stiff chamber is not linear. Filament length was determined from images every 5 min for ~ 2 h and GFP sum projections (n = 9 cells). C) Filament extension rate is substantially reduced as chamber fills up. Initial (filament length 10-20 μm) and final (filament length > 20 μm) extension rates were determined from fits to 6 × 5 min GFP sum projections. (colors represent individual cells). Bars indicate SD and **** p < 0.0001. Figure S5. Distribution of active Cdc42 is not altered during invasive growth. A) Schematic indicating fluorescence signal over the filament long axis. Quantitation of slope of Gaussian farthest from tip in red (Max Slope, in relative units), distance [file 12915_2020_833_MOESM1_ESM.pdf]
