# Supplementary material for: Mechanical force-induced morphology changes in a human fungal pathogen
Source: BMC Biol. 2020 Sep 11;18:122. doi: 10.1186/s12915-020-00833-0 (PMC7488538; doi:10.1186/s12915-020-00833-0)
Supplement: Supplementary file 4 — Additional file 4: Table S1. Strains used in the study [61, 62]. Table S2. Oligonucleotides used in the study. Table S3. Synthesized DNA used in the study. [file 12915_2020_833_MOESM4_ESM.docx]

**Table S1: Strains used in the study.**

| STRAIN | RELEVANT GENEOTYPE | REFERENCE |
| --- | --- | --- |
| BWP17 | *ura3Δ::λimm434/ura3Δ::λimm434 his1Δ::hisG/his1Δ::his arg4::hisG/arg4Δ::hisG* | [61] |
| PY2165 | Same as BWP17 with  *RP10::ARG4*-*ACT1p-GFP-RID-ADH1t* | [37] |
| PY2263 | Same as BWP17 with  *RP10::ARG4*-*ACT1p*-*CRIB-GFP-ADH1t* | [37] |
| PY3113 | Same as BWP17 with  *RP10::ARG4*-*ADH1p*-*GFP-Ct_Rac1_-ACT1t* | [32] and this study |
| PY4861 | *ura3Δ::λ imm434/ura3Δ::λ imm434 his1::hisG/HIS1::his1::hisG arg4::hisG/URA3::ARG4::arg4::hisG* | [62] |
| PY5127 | Same as PY4489 with  *NEUT5L::URA3*-*ADH1p-mScarlet-Ct_Rac1_-ACT1t* | This study |
| PY5184 | PY2263 with *ADH1p::mScarlet-URA3* | This study |

**Table S2: Oligonucleotides used in the study.**

| Primer | Sequence |
| --- | --- |
| GA3CamScarPstIp | gtactgcagGGTGCTGGCGCAGGTGCTgtttcaaaaggtgaagctg |
| CamScarletmAscI | cggcgcgccTTTATATAATTCATCCATACCACC |
| yemChmCtRacMluI | cttctagtatacgcgtcttataatatagtacattttttagctctcttaatttttcttttcttTTTATATAATTCATCCATACCACC |
| CaADH1KIxFP_S1 | ccagaattatttttttttcatcagtttaacaacaacaaacgttattgtcatacaacaacaacaacaaatacaaaaacaattatgGGTGCTGGCGCAGGTGCT |
| CaADH1KIxFP_S2 | ctgggtaatccttgtagactaattgaccaccattggtatcaaagacaacggctttttgagtttttgggatttgttcagacatTCTGATATCATCGATGAATTCGAG |

**Table S3: Synthesized DNA used in the study.**

| DNA | Sequence |
| --- | --- |
| CamScarlet | ATGGTTTCAAAAGGTGAAGCTGTTATTAAAGAATTTATGAGATTTAAAGTTCATATGGAAGGTTCAATGAATGGTCATGAATTTGAAATTGAAGGTGAAGGTGAAGGTAGACCATATGAAGGTACTCAAACTGCTAAATTAAAAGTTACTAAAGGTGGTCCATTACCATTTTCATGGGATATTTTATCACCACAATTTATGTATGGTTCAAGAGCTTTTACTAAACATCCAGCTGATATTCCAGATTATTATAAACAATCATTTCCAGAAGGTTTTAAATGGGAAAGAGTTATGAATTTTGAAGATGGTGGTGCTGTTACTGTTACTCAAGATACTTCATTAGAAGATGGTACTTTAATTTATAAAGTTAAATTAAGAGGTACTAATTTTCCACCAGATGGTCCAGTTATGCAAAAAAAAACTATGGGTTGGGAAGCTTCAACTGAAAGATTATATCCAGAAGATGGTGTTTTAAAAGGTGATATTAAAATGGCTTTAAGATTAAAAGATGGTGGTAGATATTTAGCTGATTTTAAAACTACTTATAAAGCTAAAAAACCAGTTCAAATGCCAGGTGCTTATAATGTTGATAGAAAATTAGATATTACTTCACATAATGAAGATTATACTGTTGTTGAACAATATGAAAGATCAGAAGGTAGACATTCAACTGGTGGTATGGATGAATTATATAAATAA |
